# Efficacy and Safety of Different Neoadjuvant Treatment Regimens in Locally Advanced Squamous Head and Neck Cancer

**DOI:** 10.1002/cnr2.70447

**Published:** 2026-01-26

**Authors:** Ya‐ting Ding, Bin‐bin Fang, Lian‐bing Zhu, Ke‐jin Qiu, Li‐li Yang, Hui Ye, Yun‐xia Lv, Geng‐ming Cai

**Affiliations:** ^1^ School of Clinical Medicine, Fujian Medical University Fuzhou China; ^2^ Department of Otolaryngology‐Head and Neck Surgery People's Hospital Affiliated of Quanzhou Medical College Quanzhou China; ^3^ School of Physical Education, Quanzhou Normal University Quanzhou China; ^4^ Department of Otolaryngology‐Head and Neck Surgery Xiamen Medical College Affiliated Haicang Hospital, The Sixth Hospital of Xiamen City, The Haicang Hospital of Xiamen Xiamen China; ^5^ Department of Thyroid Surgery The Second Affiliated Hospital of Nanchang University Nanchang China

**Keywords:** LA‐HNSCC, neoadjuvant therapy, network meta analysis, ORR, OS, PFS, SAEs

## Abstract

**Background:**

Head and neck squamous cell carcinoma (HNSCC), which constitutes approximately 90% of all head and neck cancers, represents a significant global health burden. A concerning trend is the rising incidence, particularly among younger populations, which has been partly attributed to human papillomavirus (HPV) infection. While treatment outcomes are favorable for early‐stage disease, approximately 60% of patients are diagnosed with locally advanced HNSCC (LA‐HNSCC), for whom prognosis remains suboptimal. Induction chemotherapy, such as the TPF regimen, is often used to reduce tumor burden but is limited by substantial toxicity. Currently, no global consensus exists on the optimal neoadjuvant approach for LA‐HNSCC. This network meta‐analysis aims to comprehensively compare the efficacy and safety of available neoadjuvant therapies for LA‐HNSCC, excluding nasopharyngeal carcinoma due to distinct treatment paradigms, in order to inform clinical decision‐making.

**Aim:**

To compare the efficacy and safety of different neoadjuvant treatment options in locally advanced head and neck squamous cell carcinoma (LA‐HNSCC).

**Methods and Results:**

We conducted a comprehensive literature search across four major databases (PubMed, Web of Science [WOS], Embase, and Cochrane Library) from their inception through August 2024. The primary outcome measures included objective response rate (ORR), overall survival (OS), progression‐free survival (PFS), and serious adverse events (SAEs). This analysis included 23 studies (19 randomized controlled trials [RCTs] and 4 non‐randomized studies [NRS]) involving 4052 patients. Network meta‐analysis (NMA) revealed the following findings: Regarding efficacy, hyperthermia + chemotherapy demonstrated superior outcomes in both ORR and OS, followed by immunotherapy + chemotherapy. Hyperthermia + chemotherapy showed significantly better ORR compared to targeted therapy + chemotherapy, TP, PF, T, and single‐agent immunotherapy (*p* < 0.05). Similarly, immunotherapy + chemotherapy outperformed all other therapies except hyperthermia + chemotherapy in ORR (*p* < 0.05). For OS, both hyperthermia + chemotherapy and TPF were significantly more effective than PF and TP (*p* < 0.05). In PFS, immunotherapy + chemotherapy showed the best results, followed by targeted therapy + chemotherapy, with immunotherapy + chemotherapy being significantly superior to PF (*p* < 0.05). Regarding safety, hyperthermia + chemotherapy showed the poorest safety profile, followed by TPF. Specifically, T and TP demonstrated significantly better safety than hyperthermia + Chemotherapyy (*p* < 0.05).

**Conclusion:**

In the neoadjuvant treatment of LA‐HNSCC, both hyperthermia + chemotherapy and immunotherapy + chemotherapy regimens have demonstrated promising therapeutic efficacy; however, their safety profiles require further comprehensive evaluation.

**Trial Registration:** PROSPERO CRD42024571174

## 1 Introduction

Head and neck squamous cell carcinoma (HNSCC) is a heterogeneous group of tumors that arise from the squamous epithelium of the oral cavity, oropharynx, larynx, and hypopharynx. It accounts for 90% of all head and neck cancer (HNC) cases diagnosed in patients [1]. According to GLOBOCAN 2020 [2], compiled by the International Agency for Research on Cancer (IARC), the latest global incidence and mortality rates for 36 cancers across 185 countries show that HNSCC cases exceed 600 000 annually, making it the seventh most common cancer worldwide and the eighth leading cause of cancer‐related mortality.

Over a 27‐year period (1990–2017) [3], the global incidence of HNSCC, particularly oral and hypopharyngeal cancers, has risen significantly, with oral cancer showing the most pronounced increase. The primary affected groups include men, individuals from low‐ and middle‐income regions, women who smoke, and younger populations. The rising incidence of HNSCC among young people has been linked to the increasing prevalence of human papilloma virus (HPV) infections [4].

Following the 2019 data update, it was observed that mortality rates for laryngeal and nasopharyngeal cancers have declined over the past three decades. However, mortality rates for other pharyngeal cancers, including oral cancer, continue to rise [5, 6, 7]. Projections indicate that the number of new oral cancer cases and related deaths is expected to increase by more than 1.5‐fold over the next 20 years [6, 8].

An analysis of survival rates among HNSCC patients revealed that laryngeal cancer has the highest 5‐year survival rate (59%), while hypopharyngeal cancer has the lowest (25%) [9]. Given the anticipated rise in HNSCC incidence, reducing mortality through optimized treatment strategies remains a critical focus.

Early‐stage HNSCC is primarily treated with surgery or radiotherapy, and the overall prognosis is favorable, with a 5‐year survival rate ranging from 60% to 95% [10]. However, approximately 60% of HNSCC patients are diagnosed at a locally advanced stage. Currently, the Chinese Society of Clinical Oncology (CSCO) recommends considering induction chemotherapy with the docetaxel, cisplatin, and 5‐fluorouracil (TPF) regimen for patients with LA‐HNSCC [11]. This approach aims to reduce tumor burden, achieve downstaging, improve surgical resection rates, and preserve organ function by increasing pathological response rates and reducing the risk of distant metastasis [12, 13, 14, 15, 16]. Despite these benefits, toxicity remains the most significant limitation of this regimen [17, 18].

Although induction chemotherapy has shown some efficacy in clinical practice, recent studies suggest that anti‐EGFR targeted combination therapy, specifically the TPC regimen (docetaxel, cisplatin, and cetuximab), which replaces 5‐fluorouracil (5‐FU) with cetuximab, may be more effective and better tolerated [19]. This makes it a promising alternative to traditional induction therapy. Schell et al. [20] supported this perspective in their research. Additionally, clinical studies have found that immune checkpoint inhibitors (ICIs) combined with anti‐EGFR‐targeted therapies are also effective in treating HNSCC [21]. However, as of now, no direct comparisons have been made with other treatment regimens. With the recent approvals of ICIs, several clinical trials have been conducted. Notably, two phase II clinical trials [22, 23] have demonstrated the efficacy and safety of pembrolizumab in LA‐HNSCC. Furthermore, the CheckMate‐358 phase I/II clinical trial [24] has shown that nivolumab is both effective and safe for treating LA‐HNSCC.

At this stage, global authoritative guidelines have not yet provided clear recommendations for neoadjuvant therapy in LA‐HNSCC, and the value of various neoadjuvant therapy regimens for LA‐HNSCC remains controversial. Therefore, this study aims to integrate data on the efficacy and safety of existing neoadjuvant therapeutic regimens for LA‐HNSCC patients through network meta‐analysis (NMA), with the goal of offering a reference for clinical treatment. The National Comprehensive Cancer Network (NCCN) guidelines highlight distinct therapeutic approaches for nasopharyngeal carcinoma compared to other HNSCCs. Given these differences in recommended treatment strategies, nasopharyngeal carcinoma was excluded from the current analysis.

## 2 Materials and Methods

### 2.1 Data Sources

In this study, we conducted a comprehensive search of all relevant articles and references published in four databases: PubMed, Web of Science (WOS), Embase, and the Cochrane Library. Additionally, the reference lists of relevant studies and reviews were reviewed to identify further information. The search strategy for the databases was modeled after the PubMed approach (Table 1), and the search period spanned from the inception of each database to August 1, 2024.

**TABLE 1:** PubMed search strategy.

	Title/abstract
#1	head and neck OR oral* OR mouth OR tongue OR buccal mucosa OR alveolar ridge OR retromolar trigone OR gingiv* OR palate OR pharyn* OR tonsil OR posterior pharyngeal wall OR soft palate OR hypopharynx* OR laryn* OR ethmoid sinus OR maxillary sinus
#2	squamous cell carcinoma
#3	#1 AND #2
#4	HNSCC
#5	#3 OR #4
#6	neoadjuvant* OR preopera* OR induction chemotherapy OR immunothera* OR target
#7	randomized controlled trial OR placebo OR RCT
#8	#5 AND #6 AND #7

### 2.2 Inclusion Criteria

1. Pathologically confirmed LA‐HNSCC: Patients must have a pathological diagnosis of LA‐HNSCC.
2. Pre‐surgical or definitive treatment requirement: The trial must include a pre‐surgical preoperative treatment, definitive pre‐treatment, or a combined treatment regimen as the intervention.
3. Two‐arm trial design: The trial must be at least a two‐arm study.
4. Outcome metrics: The trial must report the following outcome measures considered in this study:
  - Objective response rate (ORR): The proportion of patients achieving a complete or partial response after neoadjuvant therapy.
  - Serious adverse events (SAEs): Defined as the incidence of grade 3 or higher adverse events, as classified by the National Cancer Institute Common Terminology Criteria (CTCAE), occurring after neoadjuvant therapy. Any adverse event resulting in death, requiring hospitalization or prolonging hospitalization, being life‐threatening, or causing persistent or significant disability/functional impairment (CTC v1.0 to CTCAE v5.0).
  - Overall survival (OS): Defined as the time from the start of the study to the occurrence of death.
  - Progression‐free survival (PFS): Defined as the time from the start of the study to the first instance of tumor progression or death.
5. Study design and language: Both randomized controlled trials (RCTs) and non‐randomized studies (NRS) are eligible, provided they are published in Chinese or English.

### 2.3 Literature Risk of Bias Assessment

Two reviewers independently assessed the quality of the included studies using Cochrane recommended tools. RCTs were evaluated using the Risk of Bias (RoB) tool across seven domains (random sequence generation, allocation concealment, blinding of participants/personnel, blinding of outcome assessment, incomplete outcome data, selective reporting, and other biases), with each rated “Low”, “Unclear”, or “High” risk. Whereas for NRS, the ROBINS‐I tool was applied, which evaluates bias across seven chronological domains to assign an overall rating of “Low”, “Moderate”, “Serious”, or “Critical” risk of bias [25].

### 2.4 Data Extraction

Two researchers independently conducted the reading and screening of the literature based on the inclusion and exclusion criteria. Information was extracted from the final included studies, including: authors of the studies, publication date, country, sample size, intervention methods, outcome measures, and literature quality assessment, along with other relevant details. The screening process and data extraction were cross‐checked by the researchers. In cases of disagreement, a third party was consulted to resolve the discrepancies. For studies that did not report hazard ratios (HRs) and 95% confidence intervals (CIs) for OS and PFS, we used Engauge Digitizer 12.1 software to extract data from the survival curves in the original studies and calculate the HRs.

### 2.5 Statistical Analysis of Data

In this study, Bayesian NMA was conducted using the GeMTC package in R 4.3.2 software. The meta‐analysis was performed using the Markov Chain Monte Carlo (MCMC) method.

- For ORR and SAEs, the model was configured with 4 Markov chains, a step size of 1, and 5000 pre‐iterations for annealing, followed by 20 000 iterations to ensure robust model convergence.
- For OS, the model used 4 Markov chains with a step size of 1, 10 000 pre‐iterations for annealing, and 40 000 iterations to achieve robust convergence.
- For PFS, the model was set up with 4 Markov chains, a step size of 1, 5000 pre‐iterations for annealing, and 30 000 iterations to ensure robust convergence.

**FIGURE 1:**
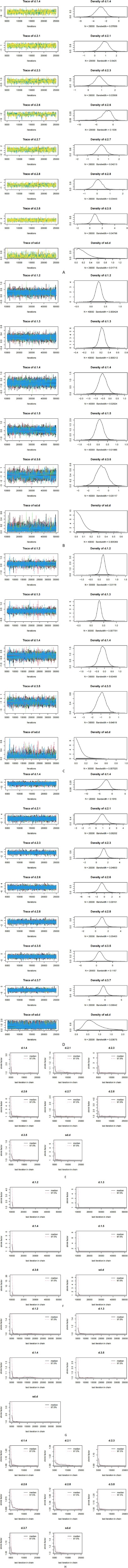
Trajectory plots, density plots, and Brooks‐Gelman‐Rubin convergence diagnostic plots for each outcome indicator. Panels A–D: Trajectory and density plots; Panels E–H: Brooks‐Gelman‐Rubin convergence diagnostic plots. Legend: ORR: Panels A and E. OS: Panels B and F. PFS: Panels C and G. SAEs: Panels D and H.

The Deviance Information Criterion (DIC) values were calculated to evaluate model performance. Convergence robustness was assessed by plotting trajectory diagrams, density diagrams, and Brooks‐Gelman‐Rubin convergence diagnostic diagrams (Figure 1). The potential scale reduction factor (PSRF) values all converged to 1, indicating satisfactory model convergence. Network evidence maps were created to visualize comparisons between different treatment options. Nodal analysis was used to assess inconsistency between direct and indirect evidence. A random‐effects model was selected to combine the data, with statistical significance set at *p* < 0.05. Heterogeneity was evaluated using the *I* ^2^ statistic, where higher values indicate greater heterogeneity. An *I* ^2^ > 50% is generally considered to indicate high heterogeneity. Effect sizes were calculated as follows:

- Dichotomous data were expressed as odds ratios (ORs).
- Survival data were expressed as hazard ratios (HRs).

Both measures were reported with 95% confidence intervals (CIs).

Treatment options were ranked based on the Surface Under the Cumulative Ranking Curve (SUCRA), with higher SUCRA values indicating better treatment efficacy. Cumulative line graphs were plotted to visualize the rankings. Publication bias was assessed using funnel plots for different outcome indicators, generated with the meta‐package in R.

### 2.6 Registration

The study protocol was registered with PROSPERO (Registration Number: CRD42024571174) prior to the initiation of the study.

## 3 Result

### 3.1 Characteristics of Included Studies

**FIGURE 2:**
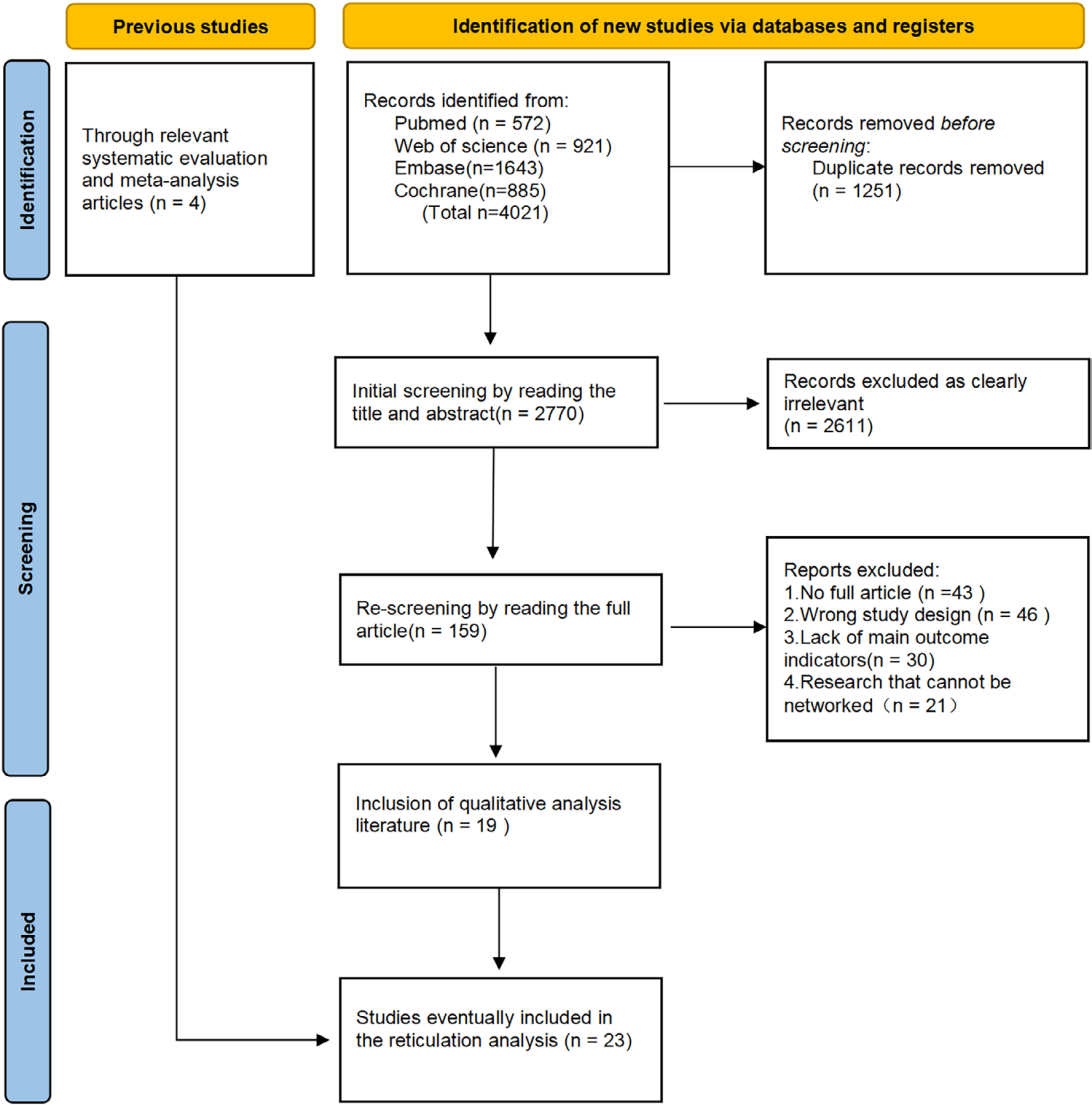
Study retrieval flowchart.

Through a comprehensive search of Chinese and English databases, 2770 documents were initially identified. After screening titles and abstracts, 2611 irrelevant documents were excluded. Following a full text review of the remaining 159 documents, 140 additional documents were excluded based on the inclusion and exclusion criteria. Ultimately, 19 documents met the eligibility requirements. Additionally, by reviewing relevant systematic reviews and meta‐analysis and conducting a gap analysis in Google Scholar, 4 new articles were identified for inclusion. In total, 23 papers were included in the study. The selection process followed the PRISMA 2020 guidelines [26], as illustrated in Figure 2.

The 23 original studies included in this analysis were all two‐arm studies [19, 27, 28, 29, 30, 31, 32, 33, 34, 35, 36, 37, 38, 39, 40, 41, 42, 43, 44, 45, 46, 47, 48]. Among these, 19 were RCTs, and 4 were NRS. Of the 22 English‐language publications, 7 studies were conducted in China. The 23 studies involved a total of 4052 patients and evaluated 8 treatment regimens:

- TPF (taxanes + platinum‐based agents + 5‐fluorouracil).
- TP (taxanes + platinum‐based agents).
- PF (platinum‐based agents +5‐fluorouracil).
- Immunotherapy (immune checkpoint inhibitors such as PD‐1/PD‐L1/CTLA‐4 or rIL‐2).
- Immunotherapy + Chemotherapy (TP/PF/TPF).
- Chemotherapy (TP/PF) + Targeted Therapy (cetuximab/tirapazamine).
- Hyperthermia + Chemotherapy (TPF).
- T (taxanes).

The basic characteristics of the included studies are detailed in Table 2.

**TABLE 2:** Basic information of the included literature.

Included studies	Region	Stage	Affected area	Sample size		Age		Treatment regimen		Intervention period	Outcome measure
				Experimental group	Control group	Experimental group	Control group	Experimental group	Control group		
Wang 2024	China	Stage III–IV without distant metastasis	Oral cavity	23	18	59	59.5	Immunotherapy + Chemotherapy	TP	3 cycles	ORR; SAEs
Noronha 2024	India	Stage IVA and IVB	Oral cavity	248	247	43 (37–49)	43 (38–52)	TP	TPF	2 cycles	SAEs; OS; PFS
Golubev 2024	Russia	Stage III–IV without distant metastasis	Oropharynx	34	34	NA	NA	TP	TPF	3 cycles	ORR
Zhang 2023	China	Stage III–IV without distant metastasis	Nasal cavity/Hypopharynx/Larynx/Oral cavity	66	62	56 (33–69)	59 (34–70)	TP	TPF	2 cycles	SAEs; OS; PFS
Wang 2023	China	Stage III–IV without distant metastasis	HNSCC	47	52	59 (34–76)	59 (34–76)	TP	Immunotherapy + Chemotherapy	3 cycles	ORR; SAEs
Chen 2022	China	Stage III–IVA	Oral cavity	34	34	NA	NA	Immunotherapy	Immunotherapy + Chemotherapy	3 cycles	ORR; SAEs
Szturz 2021	Switzerland	Stage III–IV without distant metastasis	Hypopharynx/Larynx/Oral cavity/Oropharynx	181	177	NA	NA	PF	TPF	3 cycles	OS; PFS
Ren 2021	China	Stage III–IVA	Oral cavity	60	60	NA	NA	Hyperthermia + Chemotherapy	TPF	2 cycles	ORR; SAEs; OS
Oppelt 2021	USA	Stage III–IV without distant metastasis	Oropharynx/Larynx/Hypopharynx	40	40	58 (42–78)	66 (49–84)	TP	T	2 cycles	ORR; SAEs
Keil 2021	Austria	Stage III–IVB without distant metastasis	HNSCC	49	51	58.3 ± 7.6 (40–72)	58.2 ± 8.2 (35–78)	TPF	Targeted therapy + chemotherapy	3 cycles	ORR; SAEs; OS; PFS
Sun 2020	China	Stage III–IV without distant metastasis	Hypopharynx/Larynx/Oral cavity/Oropharynx	108	111	54.96	55.7	TPF	PF	3–4 cycles	ORR; PFS
Janoray 2015	France	Stage III–IV without distant metastasis	Larynx/Hypopharynx	110	103	NA	NA	TPF	PF	3 cycles	ORR; OS
Lee 2015	Korea	Stage III–IV without distant metastasis	HNSCC	48	44	NA	NA	Targeted therapy + chemotherapy	TP	3 cycles	ORR; OS; PFS
Lakshmaiah 2015	India	Stage III–IV without distant metastasis	Hypopharynx/Larynx/Oral cavity/Oropharynx	50	50	46 (25–65)	47 (23–65)	PF	TPF	3 cycles	ORR
Huang 2011	China	Stage III–IVB without distant metastasis	Buccal/Hypopharynx/Oropharynx/Tongue	17	15	51 (36–77)	60 (37–76)	PF	TPF	3–4 cycles	ORR; PFS
Lorch 2011	USA	Stage III–IV without distant metastasis	Hypopharynx/Larynx/Oral cavity/Oropharynx	255	246	55 (38–82)	56 (33–80)	TPF	PF	3 cycles	OS; PFS
Pointreau 2009	France	Stage III–IV without distant metastasis	Larynx/Hypopharynx	110	103	57 (33–72)	56 (37–75)	TPF	PF	3 cycles	ORR; SAEs; OS
Vermorken 2007	Belgium	Stage III–IV without distant metastasis	Hypopharynx/Larynx/Oral cavity/Oropharynx	177	181	53 (31–70)	53 (30–71)	TPF	PF	4 cycles	ORR; OS; PFS
Mantovani 1998	Italy	Stage III–IV without distant metastasis	HNSCC	17	16	54.41 (38–72)	56.75 (48–66)	PF	Immunotherapy + Chemotherapy	3 cycles	ORR; SAEs; OS; PFS
Le 2006	USA	Stage IV without distant metastasis	HNSCC	29	33	59 (47–81)	56 (39–75)	PF	Targeted therapy + chemotherapy	2 cycles	ORR; OS
Hitt 2005	Spanish	Stage III–IV without distant metastasis	Oral cavity/Oropharynx/Pharynx/Larynx	193	189	55 (37–74)	56 (31–75)	PF	TPF	3 cycles	ORR; SAEs; OS
Fonseca 2005	Spanish	Stage III–IV without distant metastasis	Hypopharynx/Larynx/Oral cavity/Oropharynx	42	41	58	55	TP	PF	4 cycles	ORR; SAEs
Fietkau 2020	German	Stage III–IVB without distant metastasis	HNSCC	111	105	NA	NA	TP	PF	NA	OS

### 3.2 Quality Assessment of the Literature

**FIGURE 3:**
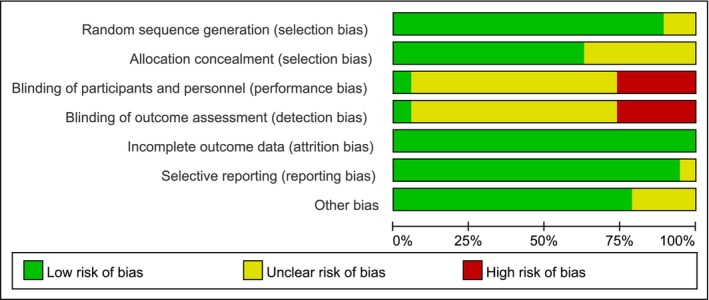
Summary of risk of bias assessment.

**FIGURE 4:**
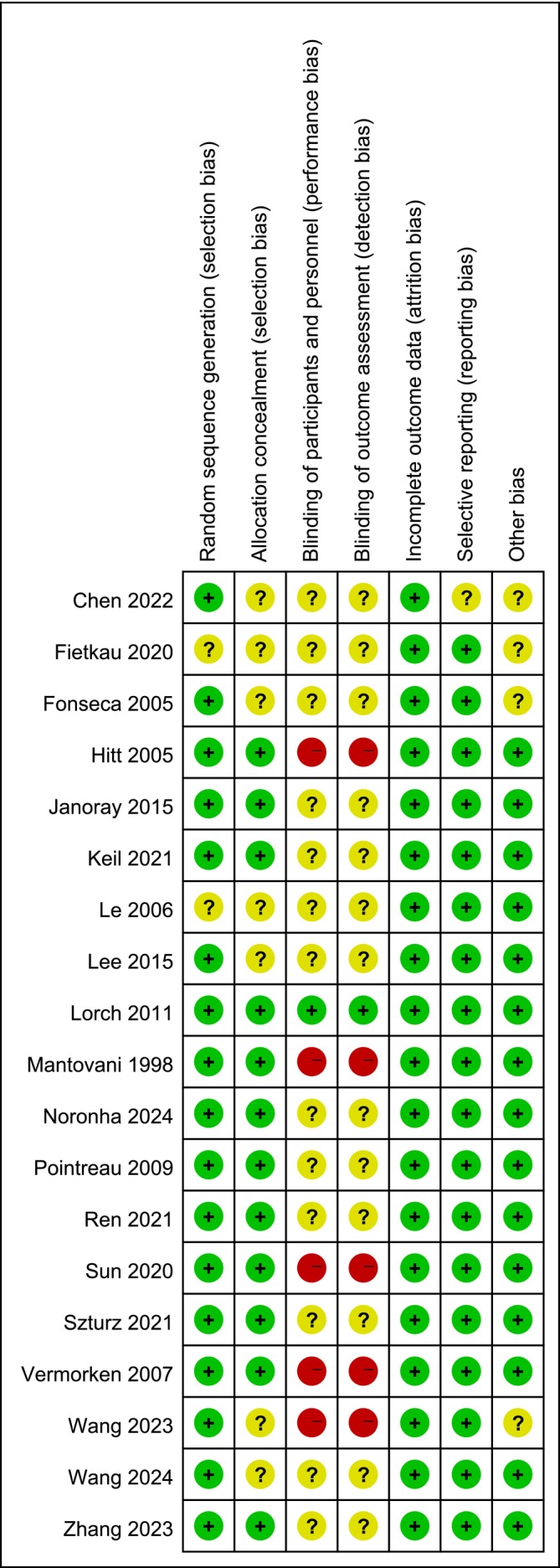
Details of the risk of bias assessment.

Nineteen of the twenty‐three papers included in this study was an RCT, and the quality of the included papers was evaluated using the Cochrane Risk of Bias Assessment Tool in RevMan 5.4 (Figure 3). The risk of bias was assessed for each study (Figure 4). In the figures, green indicates a low risk, yellow indicates an unclear risk, and red represents a high risk.

Twelve studies described the random allocation process in detail and were rated as low risk, while the remaining seven did not provide sufficient details on randomization, resulting in an unclear risk of bias. Five studies were open‐label trials and were rated as high risk, whereas the others did not provide adequate descriptions, leading to an unclear risk of bias. All studies reported their results non‐selectively and were rated as low risk in this category.

The four NRS were evaluated for risk of bias using the ROBINS‐I tool. The assessments identified one study with low risk, two with moderate risk, and one with serious risk (Table 3). All of these studies were included in the analysis.

**TABLE 3:** Consensus ROBINS‐I judgments between two reviewers by domain of bias.

	Bias due to confounding	Bias in selection of participants	Bias in measurement of interventions	Bias due to departures from intended interventions	Bias due to missing data	Bias in measurement of outcomes	Bias in selection of reports results	Overall ROBINS‐I judgment
Golubev et al. 2024	Moderate	Moderate	Low	Low	Low	Moderate	High	High
Oppelt et al. 2021	Moderate	Low	Low	Low	Low	Low	Low	Moderate
Lakshmaiah et al. 2015	Low	Low	Low	Low	Low	Low	Low	Low
Huang et al. 2011	Moderate	Moderate	Low	Low	Low	Low	Low	Moderate

## 4 Outcome Measures

### 4.1 ORR

**FIGURE 5:**
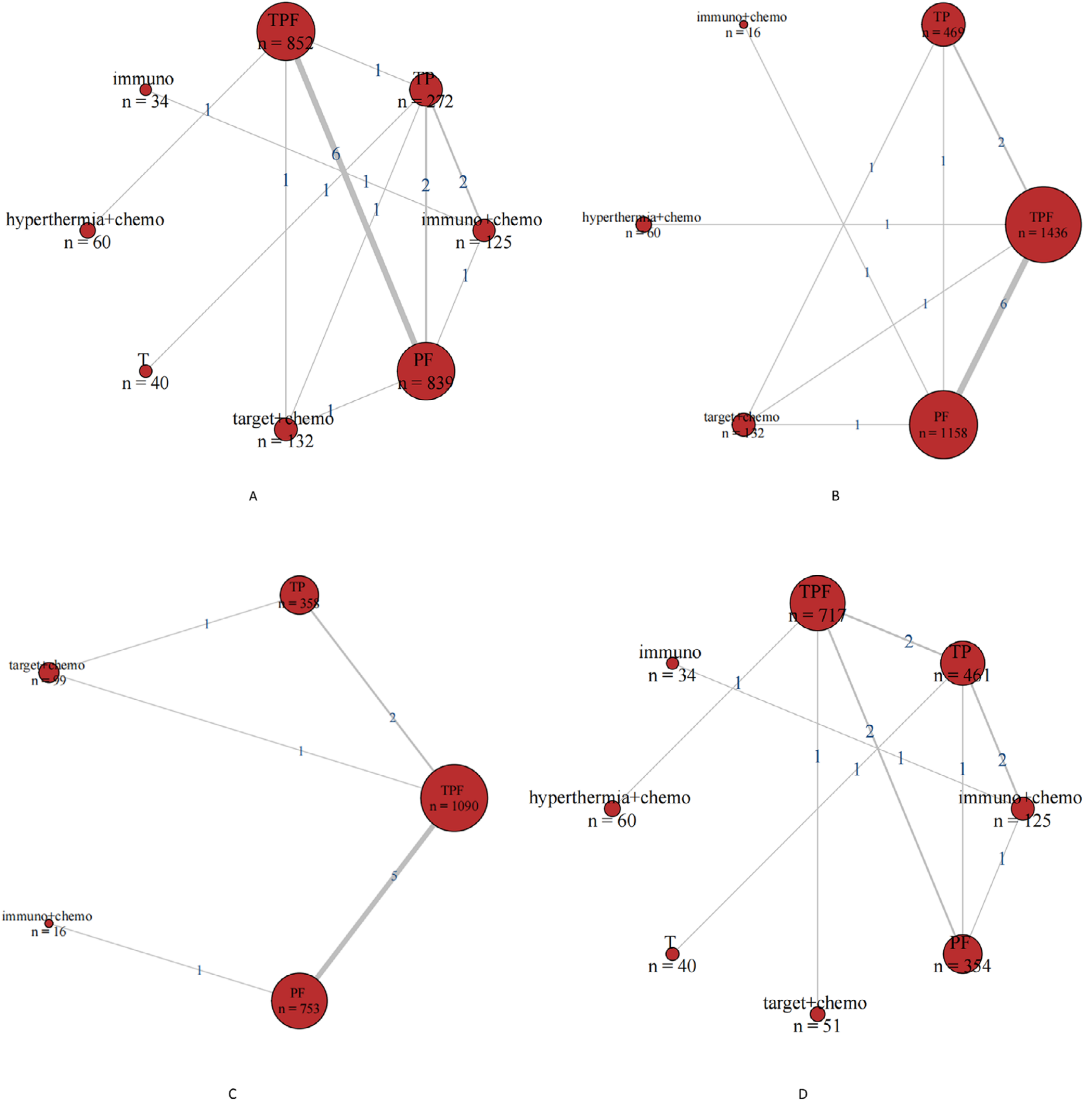
Network evidence maps (A: ORR network evidence map; B: OS network evidence map; C: PFS network evidence map; D: SAEs network evidence map). PF: Platinum‐based + 5‐fluorouracil; TPF: Taxanes + platinum‐based + 5‐fluorouracil; TP: Taxanes + platinum‐based; immuno: Immunotherapy; Immuo + Chemo: Immunotherapy + chemotherapy; target + Chemo: Targeted therapy + chemotherapy; hyperthermia + Chemo: Hyperthermia + chemotherapy; T: Taxanes.

Among the twenty‐three included papers, eighteen of which reported ORR, the literature data were extracted for network evidence mapping (Figure 5A). Each node represents a treatment option, with the size of the node corresponding to the number of patients involved. The thickness of the connecting line between two nodes, along with the associated number, indicates the number of original studies involved in the direct comparison of the two treatment options.

Through the network diagram, we can intuitively observe that five of the eight treatment options formed a closed network through direct or indirect comparisons with each other. In contrast, the remaining three treatment options did not form a closed network and could only be compared with other options through indirect comparisons. Among these, the largest number of studies involved direct comparisons between TPF and PF, with a total of three studies.

**FIGURE 6:**
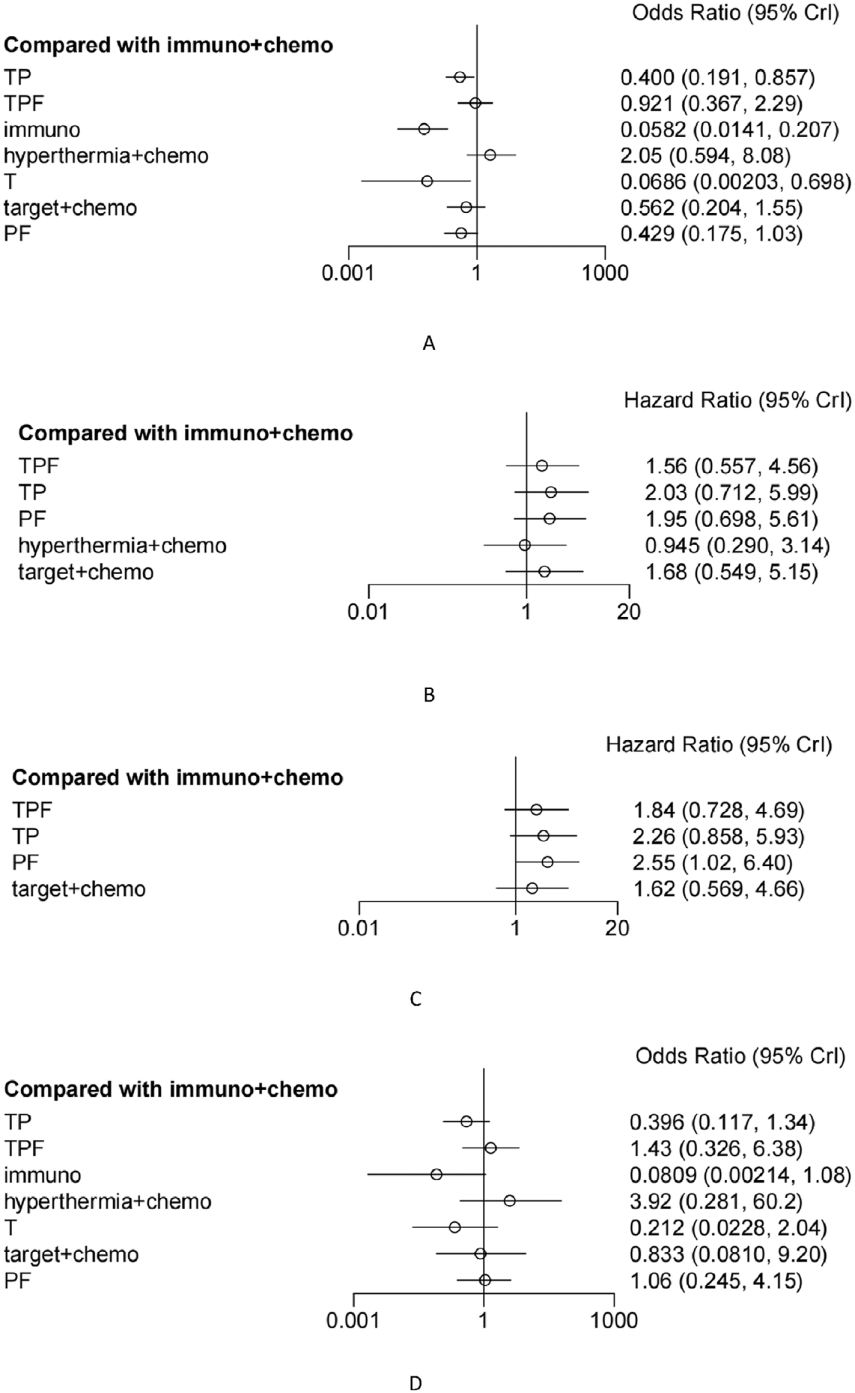
Forest plots of various indicators (A: ORR; B: OS; C: PFS; D: SAEs). Note: PF: Platinum‐based + 5‐Fluorouracil; TPF: Taxane‐based + Platinum‐based + 5‐Fluorouracil; TP: Taxane‐based + Platinum‐based; immuno: Immunotherapy; immuno + Chemo: Immunotherapy + Chemotherapy; target + Chemo: Targeted Therapy + Chemotherapy; hyperthermia + Chemo: Hyperthermia + Chemotherapy; T: Taxanes. Differences with 95% confidence intervals not crossing 1 are statistically significant (*p* > 0.05).

**FIGURE 7:**
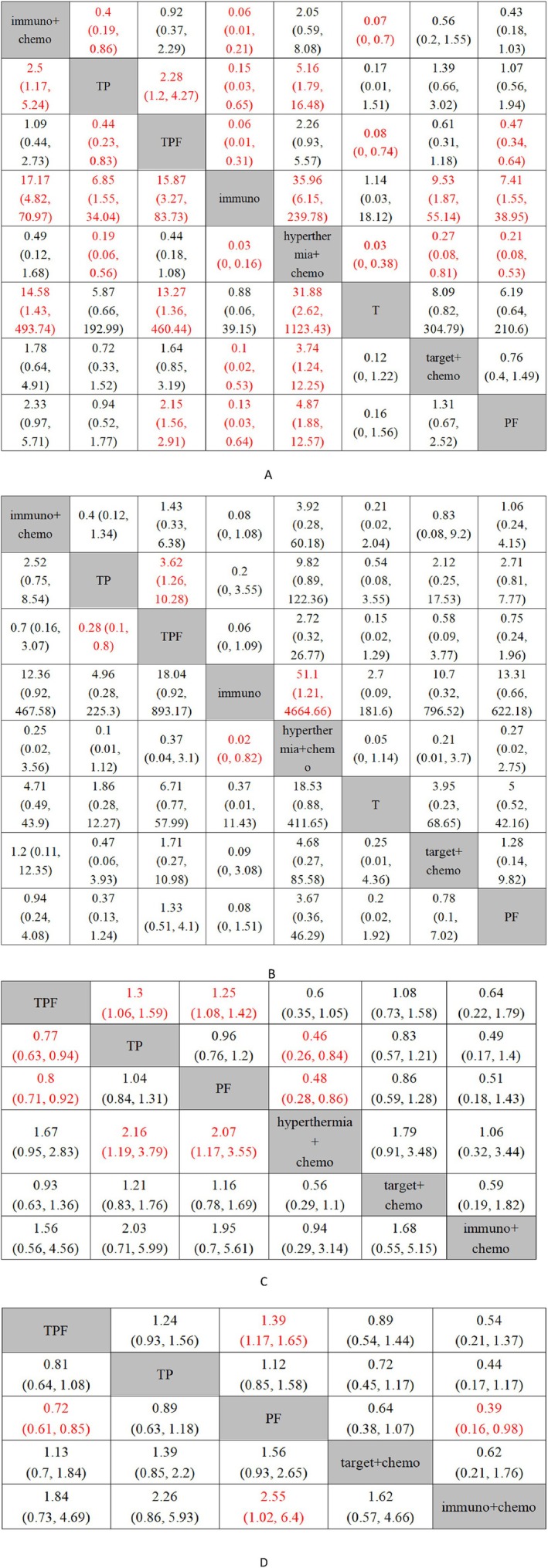
League tables of various indicators (A: ORR; B: SAEs; C: OS; D: PFS). Note: PF: Platinum‐based + 5‐Fluorouracil; TPF: Taxanes + Platinum‐based + 5‐Fluorouracil; TP: Taxanes + Platinum‐based; immuno: Immunotherapy; immuno + Chemo: Immunotherapy + Chemotherapy; target + Chemo: Targeted Therapy + Chemotherapy; hyperthermia + Chemo: Hyperthermia + Chemotherapy; T: Taxanes. Red highlighting indicates that the pairwise comparison of odds ratios (OR) is statistically significant (*p* < 0.05).

Based on the forest plot (Figure 6A) and relative effect values (Figure 7A) derived from direct and indirect comparisons of eight neoadjuvant treatment regimens across 18 studies, it was found that hyperthermia + chemotherapy (hyperthermia + TPF) significantly improved the ORR in LA‐HNSCC compared to the following treatments: Targeted therapy + chemotherapy (cetuximab + TP/TPZ + PF) (OR = 3.74 95% CI [1.24–12.25]), TP (OR = 5.16 95% CI [1.79–6.48]), PF (OR = 4.87 95% CI [1.88–12.57]), T (nab‐paclitaxel) (OR = 31.88 95% CI [2.62–1123.43]), and Mono‐immunotherapy (camrelizumab) (OR = 35.96 95% CI [6.15–239.79]). Additionally, immunotherapy + chemotherapy (toripalimab + TP/toripalimab + PF/camrelizumab + TPF/rIL‐2 + PF) demonstrated significantly better neoadjuvant efficacy compared to the other six neoadjuvant treatment regimens, excluding hyperthermia + chemotherapy, with statistical significance (*p* < 0.05).

**FIGURE 8:**
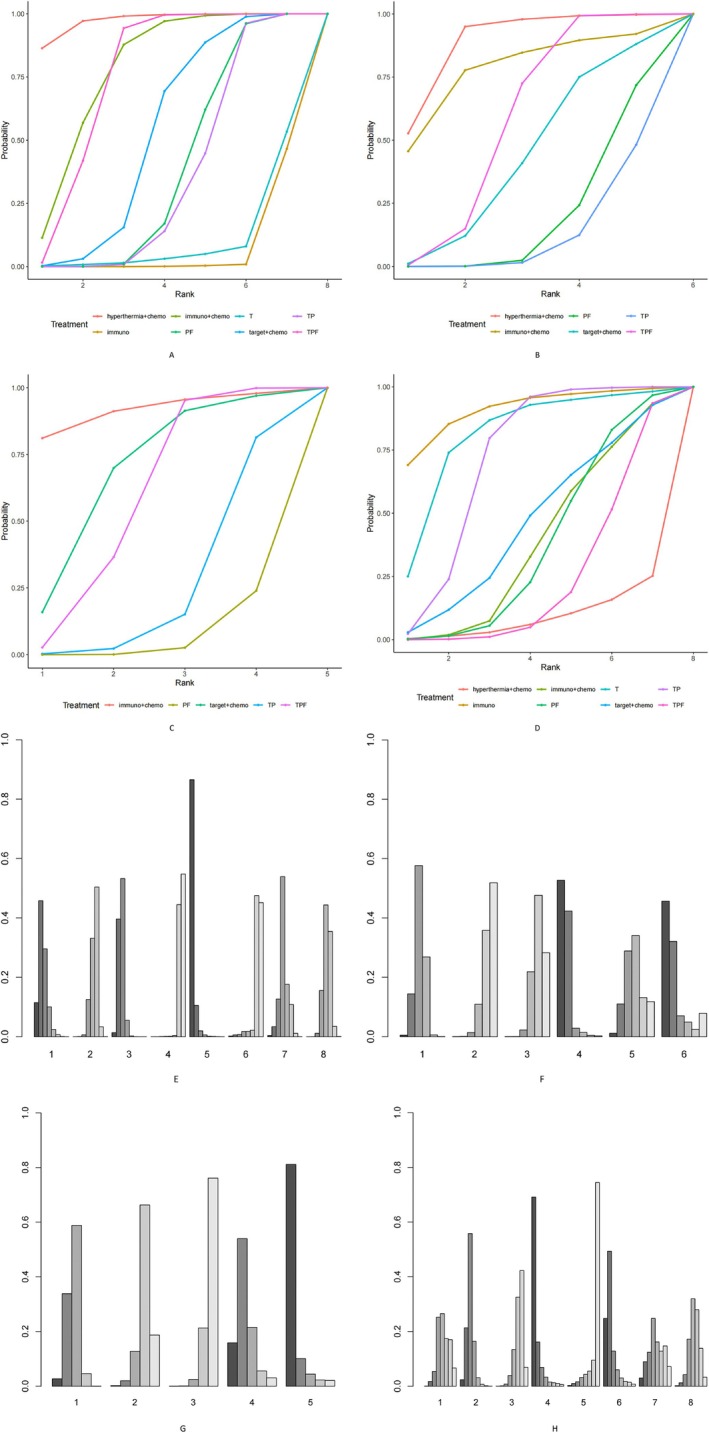
Bayesian ranking plots for various indicators (A–D: SUCRA plots; E‐H: Individual rankograms, where ORR: A, E; OS: B, F; PFS: C, G; SAEs: D, H). Note: In A‐D: PF: Platinum‐based + 5‐Fluorouracil; TPF: Taxanes + Platinum‐based + 5‐Fluorouracil; TP: Taxanes + Platinum‐based; immuno: Immunotherapy; immuno + Chemo: Immunotherapy + Chemotherapy; target + Chemo: Targeted Therapy + Chemotherapy; hyperthermia + Chemo: Hyperthermia + Chemotherapy; T: Taxanes. The numbers on the x‐axis correspond to the following neoadjuvant treatment regimens: E (ORR): 1: Immunotherapy + Chemotherapy; 2: Taxanes + Platinum‐based; 3: Taxanes + Platinum‐based + 5‐Fluorouracil; 4: Immunotherapy; 5: Hyperthermia + Chemotherapy; 6: Taxanes; 7: Targeted Therapy + Chemotherapy; 8: Platinum‐based + 5‐Fluorouracil. F (OS): 1: Taxanes + Platinum‐based + 5‐Fluorouracil; 2: Taxanes + Platinum‐based; 3: Platinum‐based + 5‐Fluorouracil; 4: Hyperthermia + Chemotherapy; 5: Targeted Therapy + Chemotherapy; 6: Immunotherapy + Chemotherapy. G (PFS): 1: Taxanes + Platinum‐based + 5‐Fluorouracil; 2: Taxanes + Platinum‐based; 3: Platinum‐based + 5‐Fluorouracil; 4: Targeted Therapy + Chemotherapy; 5: Immunotherapy + Chemotherapy. H (SAEs): 1: Immunotherapy + Chemotherapy; 2: Taxanes + Platinum‐based; 3: Taxanes + Platinum‐based + 5‐Fluorouracil; 4: Immunotherapy; 5: Hyperthermia + Chemotherapy; 6: Taxane‐based; 7: Targeted Therapy + Chemotherapy; 8: Platinum‐based + 5‐Fluorouracil.

The individual rankogram and SUCRA plot (Figure 8A,E) below present the Bayesian ranking results of different neoadjuvant treatment regimens. The SUCRA values were used to rank the eight neoadjuvant treatment options, with higher values indicating better efficacy in terms of ORR. Among the eight treatment regimens, hyperthermia + chemotherapy (hyperthermia + TPF) (SUCRA = 97.47%) had the highest SUCRA value, followed by immunotherapy + chemotherapy (toripalimab + TP/toripalimab + PF/camrelizumab + TPF/rIL‐2 + PF) (SUCRA = 78.91%).

**FIGURE 9:**
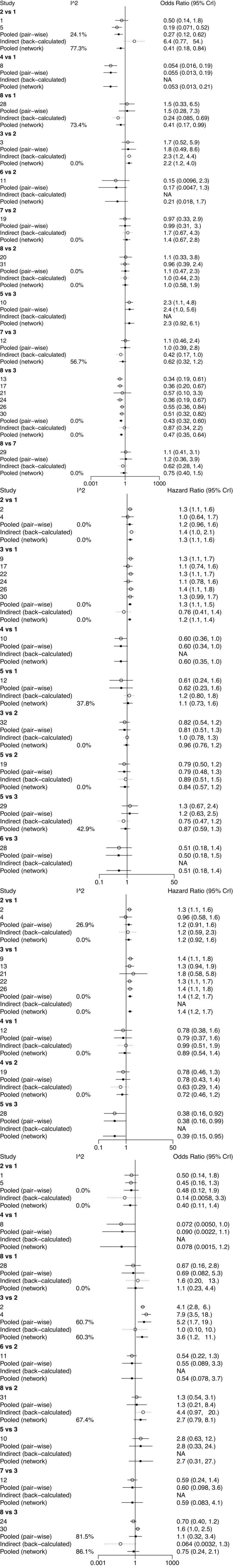
Heterogeneity tests for various indicators (A: ORR; B: OS; C: PFS; D: SAEs). Note: In each figure, the bolded numbers represent specific treatment regimens, while the non‐bolded numbers correspond to the original studies. A (ORR): 1: Immunotherapy + Chemotherapy; 2: Taxanes + Platinum‐based; 3: Taxanes + Platinum‐based +5‐Fluorouracil; 4: Immunotherapy; 5: Hyperthermia + Chemotherapy; 6: Taxanes; 7: Targeted Therapy + Chemotherapy; 8: Platinum‐based +5‐Fluorouracil. B (OS): 1: Taxanes + Platinum‐based +5‐Fluorouracil; 2: Taxanes + Platinum‐based; 3: Platinum‐based +5‐Fluorouracil; 4: Hyperthermia + Chemotherapy; 5: Targeted Therapy + Chemotherapy; 6: Immunotherapy + Chemotherapy. C (PFS): 1: Taxanes + Platinum‐based +5‐Fluorouracil; 2: Taxanes + Platinum‐based; 3: Platinum‐based +5‐Fluorouracil; 4: Targeted Therapy + Chemotherapy; 5: Immunotherapy + Chemotherapy. D (SAEs): 1: Immunotherapy + Chemotherapy; 2: Taxanes + Platinum‐based; 3: Taxanes + Platinum‐based +5‐Fluorouracil; 4: Immunotherapy; 5: Hyperthermia + Chemotherapy; 6: Taxanes; 7: Targeted Therapy + Chemotherapy; 8: Platinum‐based +5‐Fluorouracil.

Through the heterogeneity test (Figure 9A), it was found that in the comparison of ORR, there was no significant heterogeneity in the direct comparisons among various neoadjuvant treatment regimens. However, higher heterogeneity was observed in the indirect comparisons between immunotherapy + chemotherapy and TP, as well as between PF and immunotherapy + chemotherapy, with *I* ^2^ = 77.3% and *I* ^2^ = 73.4%, respectively.

### 4.2 OS

Fourteen studies reported OS, involving six neoadjuvant treatment regimens. Among these, OS‐related data from one of the three original studies were extracted from survival curves. Network comparisons revealed that the three treatment regimens formed a closed loop, while the remaining three treatment options could be indirectly compared through direct comparisons with other regimens (Figure 5B). Using Bayesian calculations, forest plots (Figure 6B) and relative effect values (Figure 7C) were obtained. Among the three neoadjuvant treatment regimens, hyperthermia + chemotherapy (hyperthermia + TPF) and TPF demonstrated significantly better efficacy as neoadjuvant treatments for LA‐HNSCC compared to PF (HR = 0.48 95% CI [0.28–0.86]; HR = 0.8 95% CI [0.71–0.92]) and TP (HR = 0.46 95% CI [0.26–0.84]; HR = 0.77 95% CI [0.63–0.94]), with statistically significant differences (*p* < 0.05).

The Bayesian ranking results for different neoadjuvant treatment regimens in the OS metric can be visually obtained from the individual rankogram (Figure 8F) and the SUCRA plot (Figure 8B). The SUCRA values derived from the data analysis indicate that hyperthermia + chemotherapy (hyperthermia + TPF) demonstrated the most favorable outcomes (SUCRA = 88.9%), followed by immunotherapy + chemotherapy (rIL‐2 + PF) (SUCRA = 77.9%). In the heterogeneity test for this metric (Figure 9B), both direct and indirect comparisons showed no significant heterogeneity, indicating good homogeneity.

### 4.3 PFS

Among the 23 original studies included, only 10 reported the PFS metric, involving five neoadjuvant treatment regimens. PFS‐related data from these two original studies were extracted from survival curves. As shown in the figure (Figure 5C), the three regimens were compared through direct and indirect connections. Data analysis yielded forest plots (Figure 6C) and relative effect values (Figure 7D). The results indicate that TPF and immunotherapy + chemotherapy (rIL‐2 + PF) significantly improved PFS in LA‐HNSCC patients compared to PF (HR = 0.72 95% CI [0.61–0.85]; HR = 0.3995% CI [0.16–0.98]), with statistically significant differences (*p* < 0.05).

Through Bayesian analysis of the extracted data, individual rankograms (Figure 8G) and SUCRA plots (Figure 8C) were generated. Based on the SUCRA values, it was found that immunotherapy + chemotherapy (rIL‐2 + PF) (SUCRA = 91.7%) provided better PFS as a neoadjuvant treatment for LA‐HNSCC patients, followed by targeted + chemotherapy (cetuximab + TP) (SUCRA = 68.5%). Both direct and indirect comparisons for this metric showed no significant heterogeneity (Figure 9C).

### 4.4 SAEs

Twelve studies reported SAEs, involving eight neoadjuvant treatment regimens. The LA‐HNSCC network diagram (Figure 5D) shows that comparisons among these four treatment regimens form a closed loop, while the remaining four regimens, which do not form a closed loop, are compared indirectly with other treatment options. Through computational analysis of the eight neoadjuvant treatment regimens, forest plots (Figure 6D) and relative effect values (Figure 7B) were obtained. The results indicate that patients receiving single‐agent immunotherapy as neoadjuvant treatment experienced significantly fewer SAEs compared to those receiving hyperthermia + chemotherapy (hyperthermia + TPF) (OR = 0.02 95% CI [0–0.82]). Additionally, TP as a neoadjuvant treatment showed significantly fewer SAEs than TPF (OR = 0.28 95% CI [0.1–0.8]), with statistically significant differences (*p* < 0.05). No statistically significant differences were observed among the other neoadjuvant treatment regimens.

Based on the individual rankograms (Figure 8H) and SUCRA plots (Figure 8D) obtained from the comparisons, the SUCRA values indicate that single‐agent immunotherapy as a neoadjuvant treatment resulted in the fewest SAEs (SUCRA = 91.05%), followed by T (SUCRA = 81.66%). In contrast, hyperthermia + chemotherapy showed the poorest performance in terms of SAEs (SUCRA = 8.83%), followed by TPF (SUCRA = 24.26%).

Through the heterogeneity test using the software (Figure 9D), it was observed that both the direct comparison (*I* ^2^ = 81.5%) and the indirect comparison (*I* ^2^ = 86.1%) between TPF and PF exhibited significant heterogeneity.

## 5 Sensitivity Analysis

After excluding studies judged to be at high risk of bias from the included literature, data analysis was performed. The results showed that although the ranking of interventions remained largely unchanged across the four outcome indicators, heterogeneity was well controlled in the analyses of ORR, SAEs, and OS (Supplement 1).

Further analysis was conducted on studies that assessed SAEs using CTCAE version 3.0 or higher. The ranking of treatment regimens in terms of SAEs showed no significant change. However, statistically significant differences were observed between TP and T plus hyperthermia combined with chemotherapy, as well as between immunotherapy and T versus TPF. Moreover, heterogeneity was moderately reduced in these comparisons (Supplement 2).

Finally, after the exclusion of NRS from the pooled analysis, the overall survival (OS) data were unaffected, and the results for the remaining three outcomes remained consistent, with no significant changes in the observed heterogeneity between endpoints (Supplement 3).

## 6 Publication Bias

**FIGURE 10:**
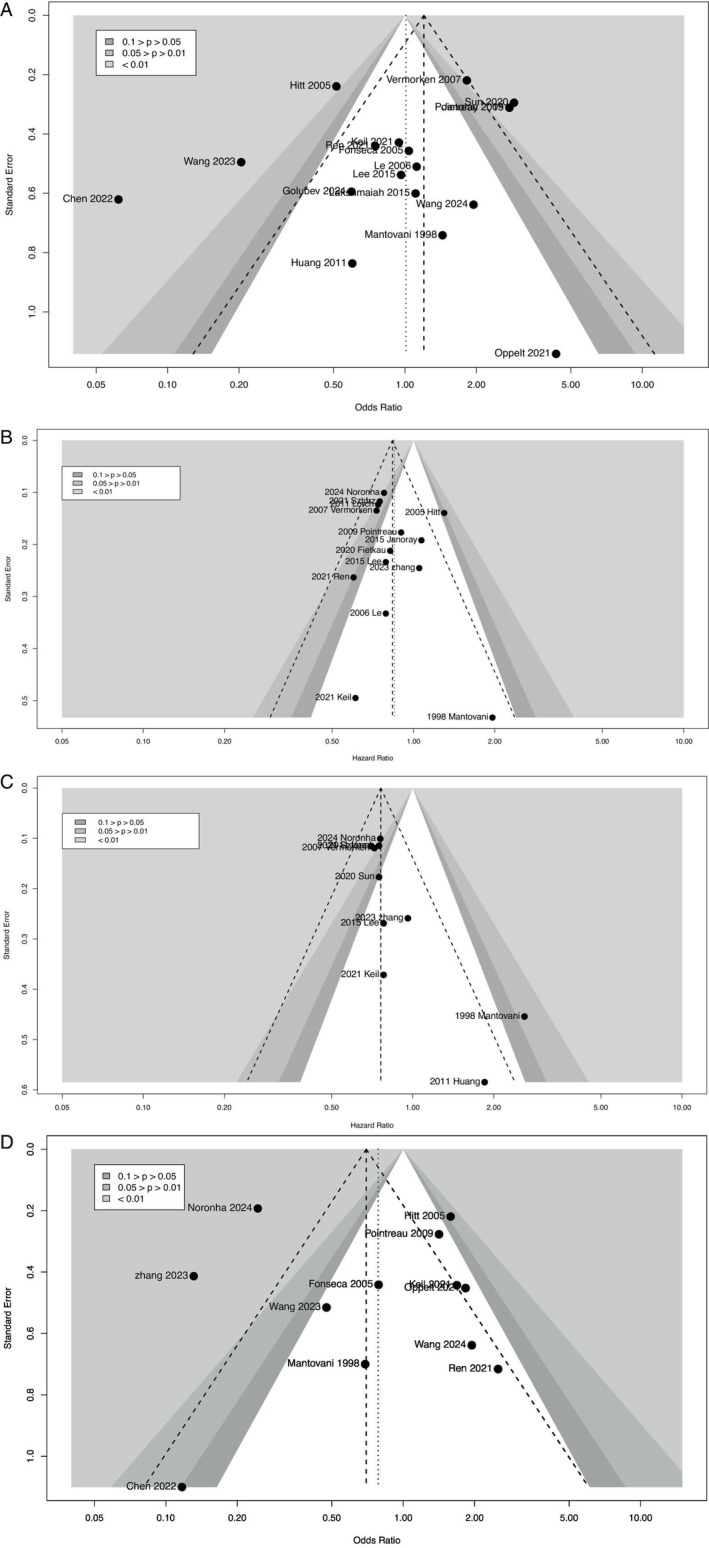
Funnel plots for publication bias of each indicator (A: ORR; B: OS; C: PFS; D: SAEs).

In this study, we conducted publication bias tests for the different indicators included, resulting in the following funnel plots (Figure 10). These funnel plots allow us to assess the presence of publication bias for each outcome indicator. According to the publication bias detection method recommended by Sterne et al. [49], for the ORR and SAEs indicators, which are binary data, we selected Peters's test. The *p*‐value for ORR after testing was 0.2759 (*p* > 0.05), and for SAEs, it was 0.5609 (*p* > 0.05), both indicating no publication bias. For the OS‐related articles, we used Egger's test for publication bias, and the resulting *p*‐value was 0.5001 (*p* > 0.05), suggesting no publication bias. However, for the PFS‐related articles, the *p*‐value was 0.0105 (*p <* 0.05), indicating potential publication bias.

## 7 Discussion

HNSCC often presents with an insidious onset and is characterized by high heterogeneity. More than 60% of patients are diagnosed at a locally advanced stage [50]. Due to the high risk of recurrence in LA‐HNSCC, the overall survival rate remains below 50%, even with multimodal treatments that include radical surgery, radiotherapy, and chemotherapy [51]. In light of this, researchers have been exploring alternative approaches for the diagnosis and treatment of LA‐HNSCC. In recent years, treatment strategies combining neoadjuvant therapy with surgery and adjuvant therapy have shown promising results, indicating the potential of neoadjuvant regimens in the future management of this disease. The increasing number of clinical studies on neoadjuvant therapy for LA‐HNSCC reflects its growing acceptance. However, existing studies on neoadjuvant therapy still lack comprehensive systematic reviews to compare the efficacy and safety of different neoadjuvant regimens, and there is no unified conclusion regarding their efficacy differences. Therefore, this study employs a network meta‐analysis to compare the effectiveness and safety of eight neoadjuvant treatment regimens across 23 included studies, aiming to provide more robust evidence for future research.

The results of this meta‐analysis indicate that among the various neoadjuvant treatment regimens for locally advanced head and neck squamous cell carcinoma (LA‐HNSCC), hyperthermia + chemotherapy (hyperthermia + TPF) and immunotherapy + chemotherapy (toripalimab + TP/toripalimab + PF/camrelizumab + TPF/rIL‐2 + PF) demonstrated superior ORR compared to induction chemotherapy alone. Additionally, neoadjuvant immunotherapy + chemotherapy involving rIL‐2 + PF showed better OS and PFS outcomes than induction chemotherapy alone. However, due to the lack of PFS data in studies on hyperthermia + chemotherapy, the efficacy of this regimen in terms of PFS could not be determined. Surprisingly, although hyperthermia + chemotherapy and TPF were associated with higher rates of severe adverse events in LA‐HNSCC patients, the difference in severe side effects was only statistically significant when comparing hyperthermia + chemotherapy with single‐agent immunotherapy (camrelizumab). No significant differences were observed when comparing hyperthermia + chemotherapy with other regimens, and TPF only showed a statistically significant difference when compared with TP. Interestingly, the clinically commonly used neoadjuvant targeted therapy + chemotherapy (cetuximab + TP) demonstrated moderate performance in terms of ORR, OS, and PFS, with no statistically significant differences compared to the top‐performing regimens. Moreover, there were no significant differences in severe adverse reactions between cetuximab + TP and the leading neoadjuvant regimens.

It is crucial to emphasize that our conclusions regarding the efficacy and safety of hyperthermia + chemotherapy are derived from a single study. Although this finding is promising, it must be interpreted with caution and is insufficient to serve as a basis for definitive clinical guidance. Future large‐scale, multi‐center trials are required to validate this result.

This study compared the efficacy and safety of different neoadjuvant therapies for patients with LA‐HNSCC using four outcome indicators: ORR, OS, PFS, and SAEs. Prior to initiating the study, a detailed search strategy was developed, and comprehensive data were included. The outcome measures from the original studies were evaluated to ensure transferability of the research. Inconsistency tests were conducted, and both direct and indirect comparisons for all outcomes showed consistency. However, substantial heterogeneity was observed in the ORR and SAEs outcomes, suggesting that treatment effects may not be uniform and could be influenced by patient characteristics or intervention details. Therefore, caution should be exercised when generalizing the findings.

Previous studies have identified hyperthermia as a promising approach in cancer treatment, demonstrating its potential to enhance the therapeutic efficacy for various types of tumors [52, 53]. Los et al. also found that hyperthermia can increase the utilization of cisplatin by tumor cells [54, 55]. Additionally, related research has highlighted the synergistic effects of hyperthermia with various chemotherapeutic agents and its ability to counteract drug resistance [55]. This study suggests a potential advantage of hyperthermia combined with chemotherapy. However, despite this observed benefit, a significant incidence of severe adverse events was reported. It is important to note that the evidence for this specific regimen was derived from only a single original study, raising the possibility of bias and imprecision. Therefore, these findings warrant validation in larger, well‐designed clinical trials. Given the current limited evidence, the results should be interpreted with caution and cannot be considered robust. Consequently, the application of hyperthermia plus chemotherapy in LA‐HNSCC should currently be approached with careful consideration.

In the context of immunotherapy combined with chemotherapy, Leduc et al. [56] conducted a retrospective experimental study and found that in patients with LA‐HNSCC, the PD‐L1 positivity rate in tumor‐infiltrating immune cells increased from 9.5% to 38% after TPF induction chemotherapy. Additionally, the density of CD8+ lymphocytes increased (237 cells/mm^2^ vs. 512 cells/mm^2^). These findings suggest that TPF induction chemotherapy may promote the activation of the PD‐L1 immune regulatory pathway, further indicating the potential of neoadjuvant immunotherapy + chemotherapy in the treatment of LA‐HNSCC. Similarly, a meta‐analysis by Chen et al.(2023) [57] demonstrated that neoadjuvant immunotherapy + chemotherapy yields a better ORR compared to neoadjuvant immunotherapy alone in the treatment of HNSCC. This enhanced efficacy is likely due to the synergistic effects of the two treatment modalities in HNSCC.

Currently, TPF and targeted therapy + chemotherapy (cetuximab + TP) are widely used in the neoadjuvant treatment of LA‐HNSCC. A retrospective study indicated that compared to TPF induction chemotherapy(ORR = 63.3%) [19], neoadjuvant targeted therapy combined with chemotherapy (ORR 74.5%) demonstrated better efficacy and tolerability, suggesting it as a viable alternative to TPF induction therapy. However, the comparative analysis in this study did not reveal the anticipated advantages of either neoadjuvant treatment regimen. Therefore, the use of these two treatment options requires more careful consideration.

## 8 Limitations

This meta‐analysis has several limitations that should be considered when interpreting the findings. First, although a network meta‐analysis allows for simultaneous direct and indirect comparisons across multiple treatments, the limited number of included studies resulted in sparse direct evidence for some comparisons and reliance solely on indirect evidence for others, which may increase the uncertainty of the results. Second, while treatment rankings were derived using Bayesian methods, the lack of statistically significant differences between certain interventions—together with clinical heterogeneity in tumor sites and patient ethnicity across studies—limits the reliability of these rankings as indicators of true therapeutic superiority. Third, some randomized trials did not adequately describe blinding or allocation concealment, and the inclusion of non‐randomized studies may introduce residual confounding due to their inherent design limitations. Moreover, the use of different CTCAE versions for adverse event reporting may have introduced heterogeneity and measurement bias. Although we performed sensitivity analyses including only studies using CTCAE v3.0 or higher, subtle differences in grading criteria between versions could still affect the precision of safety outcomes. We therefore recommend that future trials adopt a unified and up‐to‐date CTCAE version. Finally, our analysis has limitations regarding the Phase III trials. Although several were included, most only compared the TPF regimen with the PF regimen. Moreover, the remaining evidence comes from Phase II studies. It is important to note that Phase II trials are not designed to confirm efficacy and often involve different patient populations. Therefore, the current conclusions should be interpreted with caution. More definitive conclusions will require future updates with accumulating data from Phase III trials.

## 9 Conclusions

In this study, we evaluated the efficacy and safety of various neoadjuvant treatment regimens currently used for LA‐HNSCC. To date, we have conducted a comprehensive summary and assessment of neoadjuvant treatment options for LA‐HNSCC. The results indicate that hyperthermia + chemotherapy and immunotherapy + chemotherapy show more favorable outcomes in terms of ORR, OS, and PFS compared to TPF and targeted therapy + chemotherapy in the neoadjuvant treatment of LA‐HNSCC. This suggests that these two neoadjuvant treatment regimens hold greater potential in the management of LA‐HNSCC. Notably, in terms of severe adverse effects, neoadjuvant immunotherapy + chemotherapy and targeted therapy + chemotherapy demonstrated no statistically significant differences when compared to other treatment regimens, making them more advantageous. Therefore, when considering these factors, neoadjuvant immunotherapy or targeted therapy combined with chemotherapy may offer a potential benefit for patients with LA‐HNSCC. With the gradual approval of ICIs, an increasing number of clinical trials involving neoadjuvant immunotherapy + chemotherapy or dual immunotherapy are emerging. However, these studies primarily focus on advanced or recurrent/metastatic HNSCC patients. Future research should place greater emphasis on LA‐HNSCC patients to generate more data for further validation and updates to this study.

## Author Contributions

**Ya‐ting Ding:** software, formal analysis, writing – original draft, writing – review and editing. **Bin‐bin Fang:** methodology, visualization. **Lian‐bing Zhu:** data curation, writing – original draft. **Ke‐jin Qiu:** data curation, writing – original draft. **Li‐li Yang:** data curation, validation. **Hui Ye:** data curation, visualization. **Yun‐xia Lv:** writing – review and editing, supervision. **Geng‐ming Cai:** conceptualization, writing – review and editing, funding acquisition, supervision.

## Funding

This work was sponsored by Fujian Provincial Health Technology Project (No. 2024CXB023) and Science and Technology Program of Haicang District of Xiamen, China (No. 350205Z20242004).

## Conflicts of Interest

The authors declare no conflicts of interest.

## Supporting information


**Data S1:** Supplementary file 1.

**Data S2:** Supplementary file 2.

**Data S3:** Supplementary file 3.

## Data Availability

The data that support the findings of this study are available from the corresponding author upon reasonable request.
