# Supplementary material for: Efficacy and Safety of Different Neoadjuvant Treatment Regimens in Locally Advanced Squamous Head and Neck Cancer
Source: Cancer Rep (Hoboken). 2026 Jan 26;9(1):e70447. doi: 10.1002/cnr2.70447 (PMC12835624; doi:10.1002/cnr2.70447)
Supplement: Supplementary file 1 — Data S1: Supplementary file 1. [file CNR2-9-e70447-s003.docx]

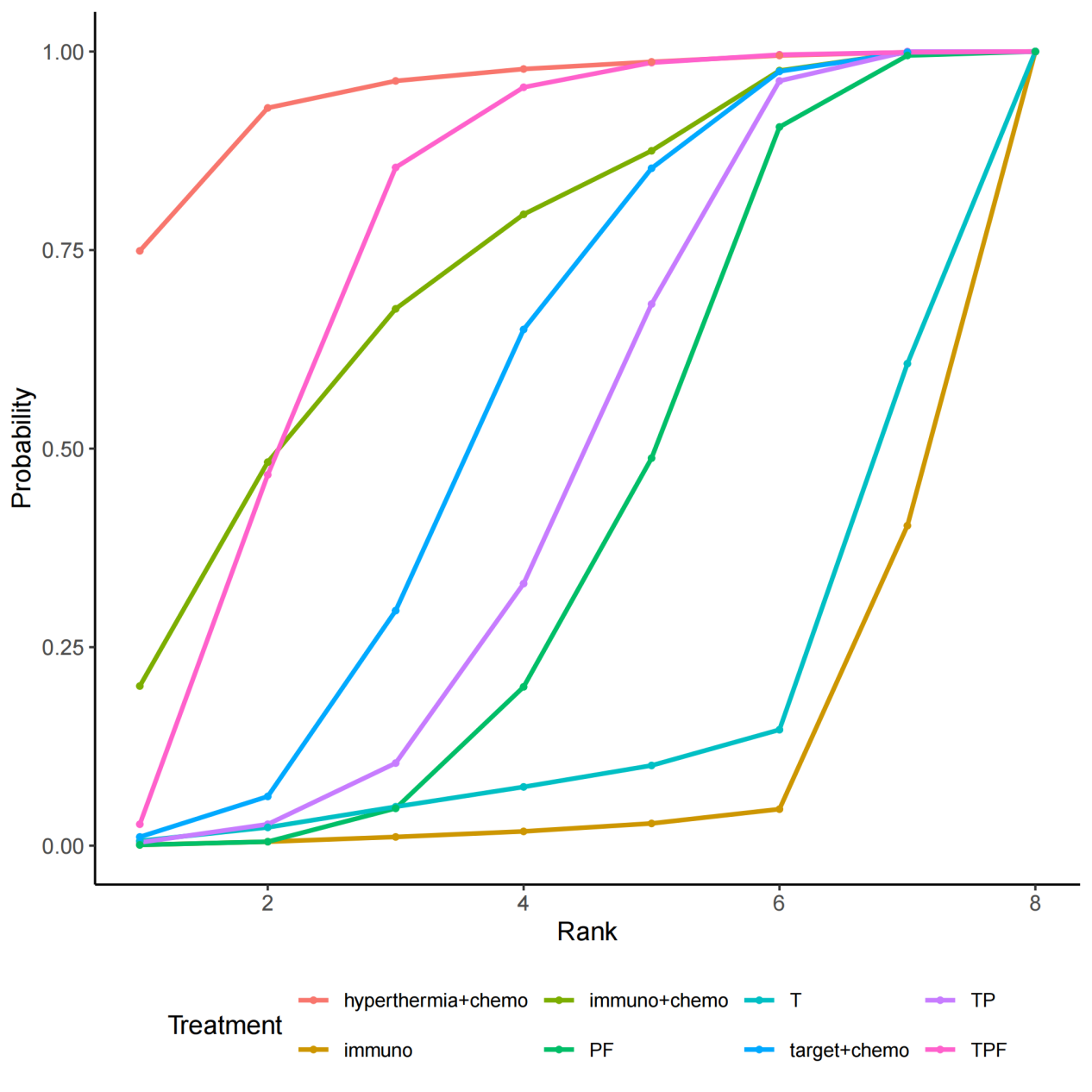


SUCRA for ORR After Excluding High Risk of Bias Studies

Note: PF: Platinum-based + 5-Fluorouracil; TPF: Taxanes + Platinum-based + 5-Fluorouracil; TP: Taxanes + Platinum-based; immuno: Immunotherapy; immuno+Chemo: Immunotherapy + Chemotherapy; target+Chemo: Targeted Therapy + Chemotherapy; hyperthermia+Chemo: Hyperthermia + Chemotherapy; T: Taxanes


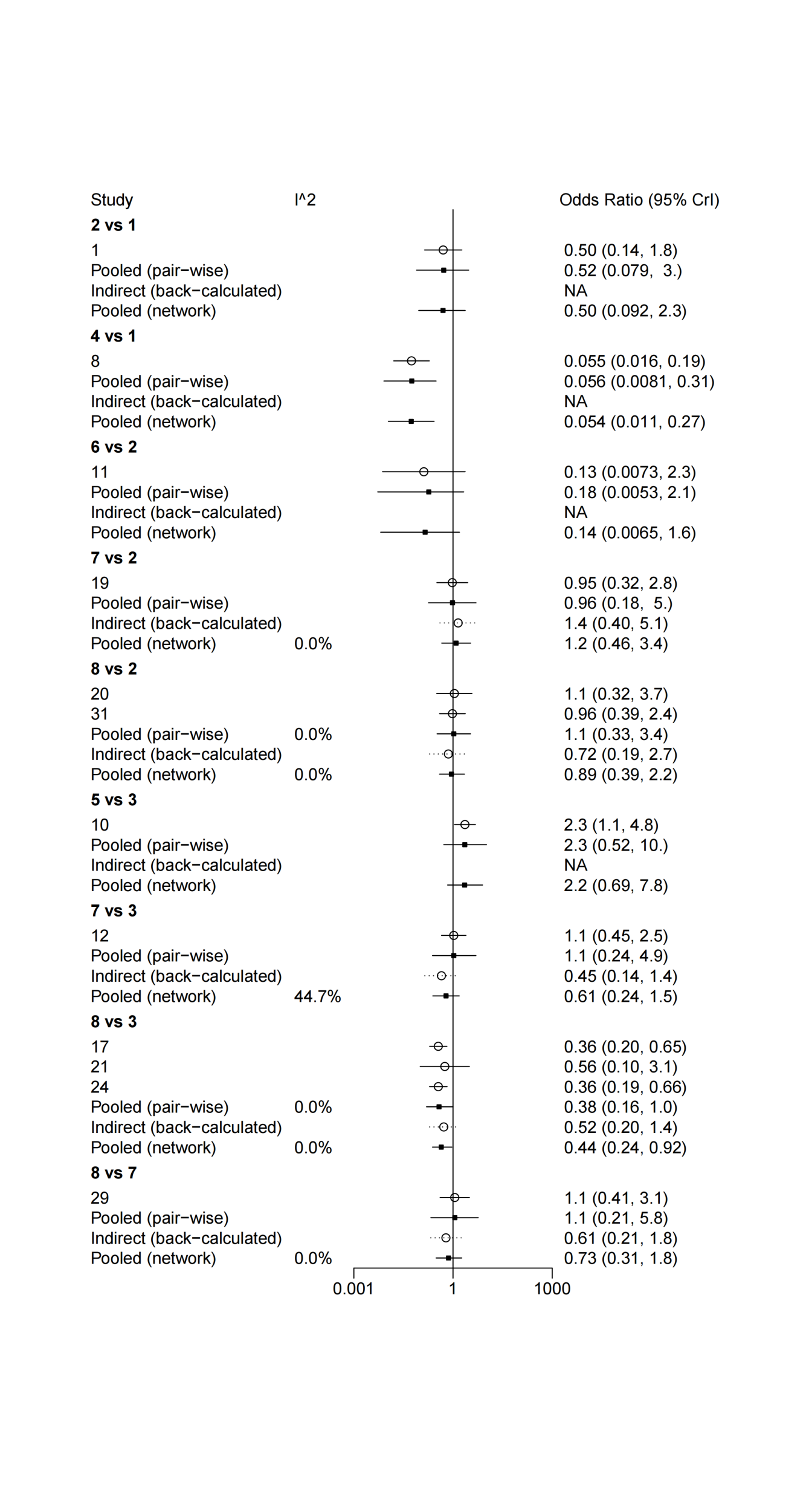


Heterogeneity of ORR After Excluding High Risk of Bias Studies

Note: In each figure, the bolded numbers represent specific treatment regimens, while the non-bolded numbers correspond to the original studies. 1: Immunotherapy + Chemotherapy; 2: Taxanes + Platinum-based; 3: Taxanes + Platinum-based + 5-Fluorouracil; 4: Immunotherapy; 5: Hyperthermia + Chemotherapy; 6: Taxanes; 7: Targeted Therapy + Chemotherapy; 8: Platinum-based + 5-Fluorouracil.


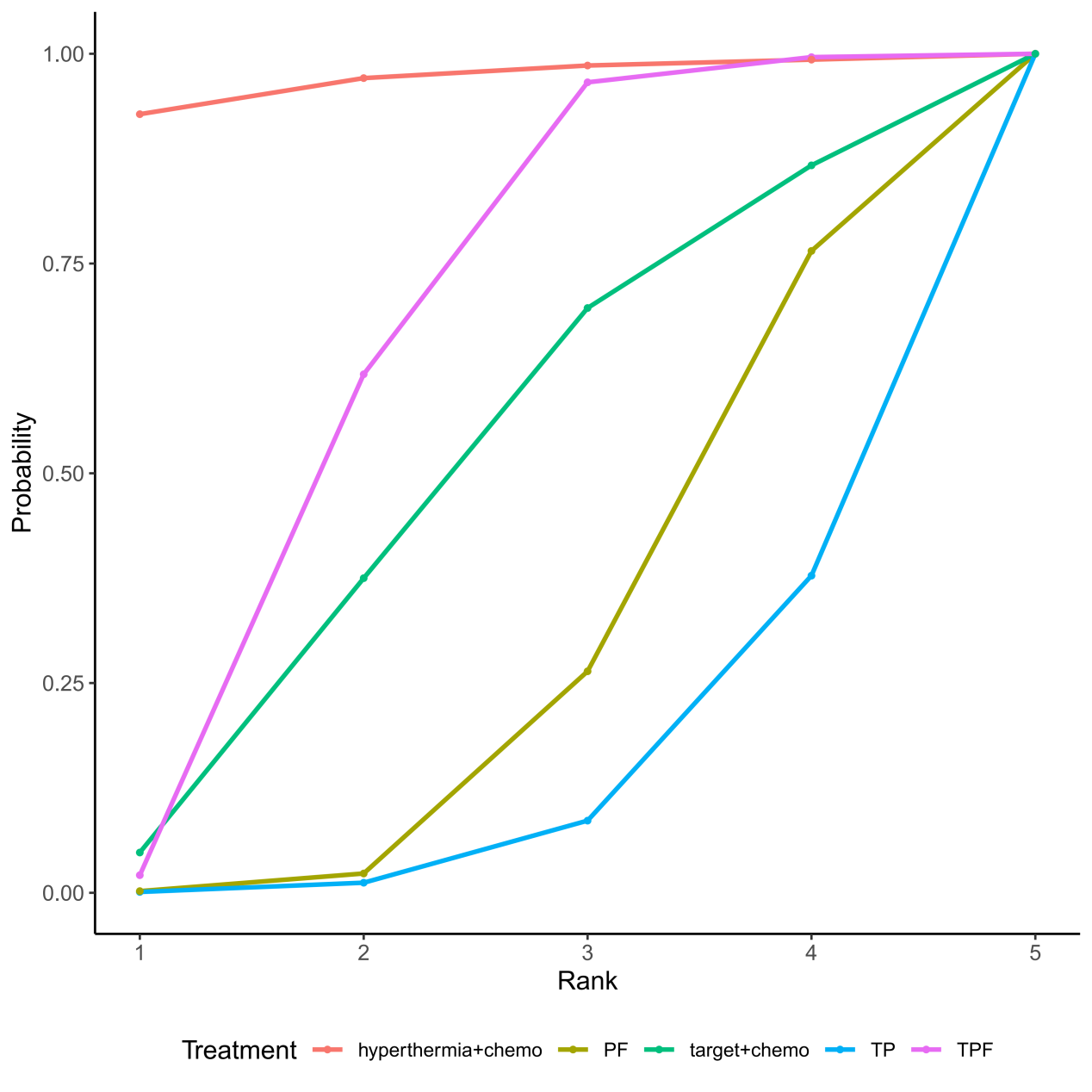


SUCRA for OS After Excluding High Risk of Bias Studies

Note: PF: Platinum-based + 5-Fluorouracil; TPF: Taxanes + Platinum-based + 5-Fluorouracil; TP: Taxanes + Platinum-based; target+Chemo: Targeted Therapy + Chemotherapy; hyperthermia+Chemo: Hyperthermia + Chemotherapy.


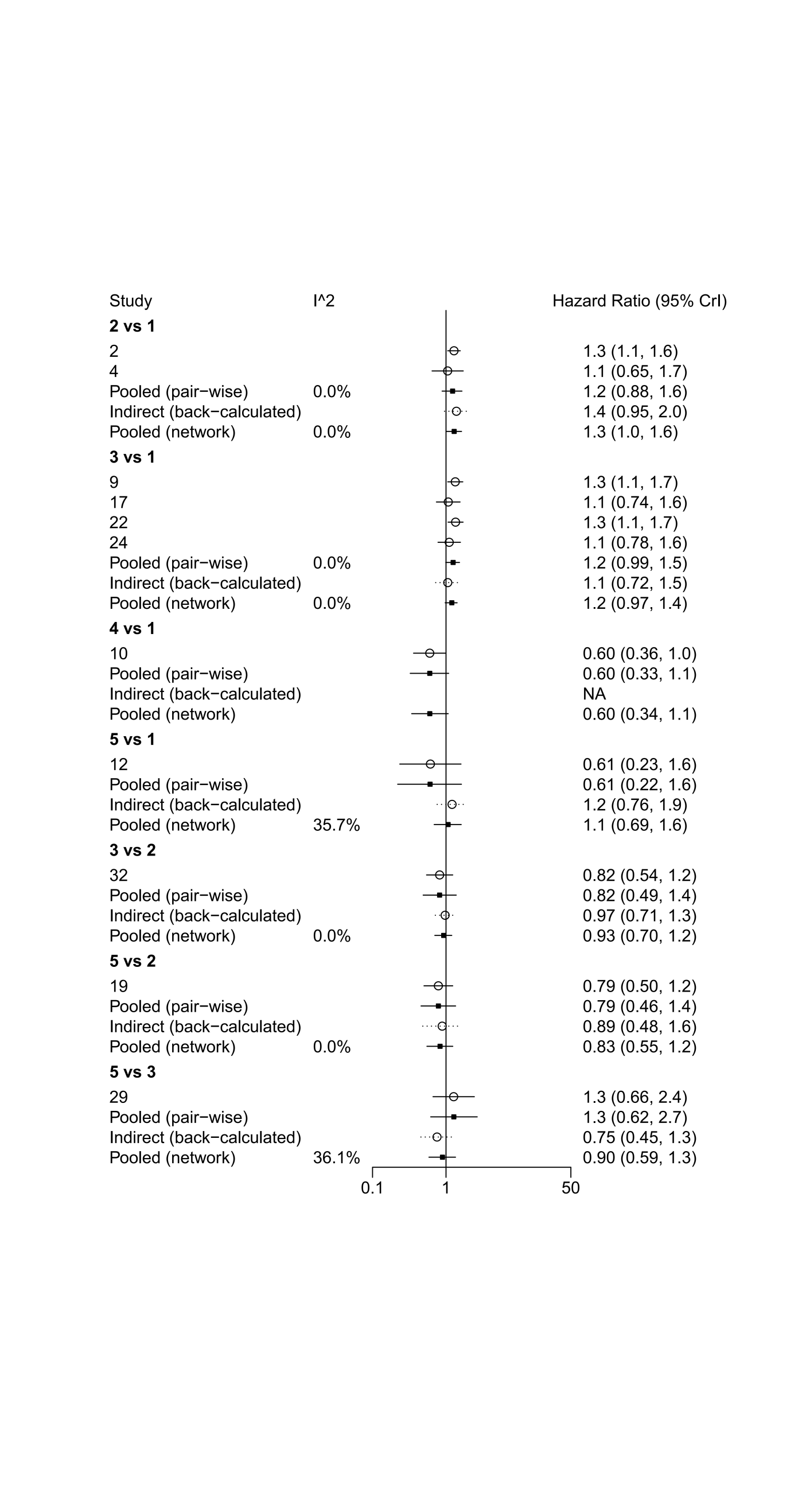


Heterogeneity of OS After Excluding High Risk of Bias Studies

Note: In each figure, the bolded numbers represent specific treatment regimens, while the non-bolded numbers correspond to the original studies. 1: Taxanes + Platinum-based + 5-Fluorouracil; 2: Taxanes + Platinum-based; 3: Platinum-based + 5-Fluorouracil; 4: Hyperthermia + Chemotherapy; 5: Targeted Therapy + Chemotherapy; 6: Immunotherapy + Chemotherapy.


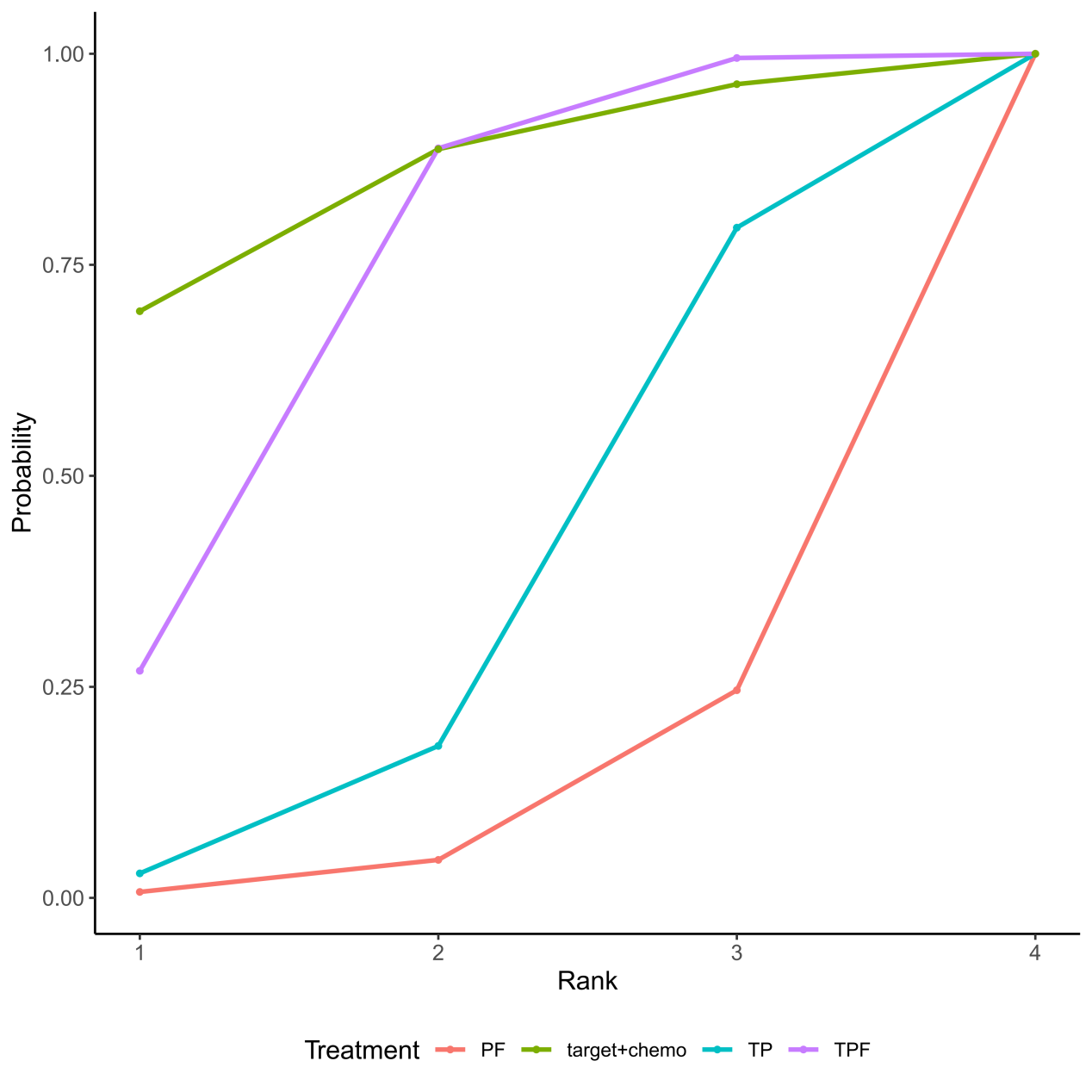


SUCRA for PFS After Excluding High Risk of Bias Studies

Note: PF: Platinum-based + 5-Fluorouracil; TPF: Taxanes + Platinum-based + 5-Fluorouracil; TP: Taxanes + Platinum-based; target+Chemo: Targeted Therapy + Chemotherapy.


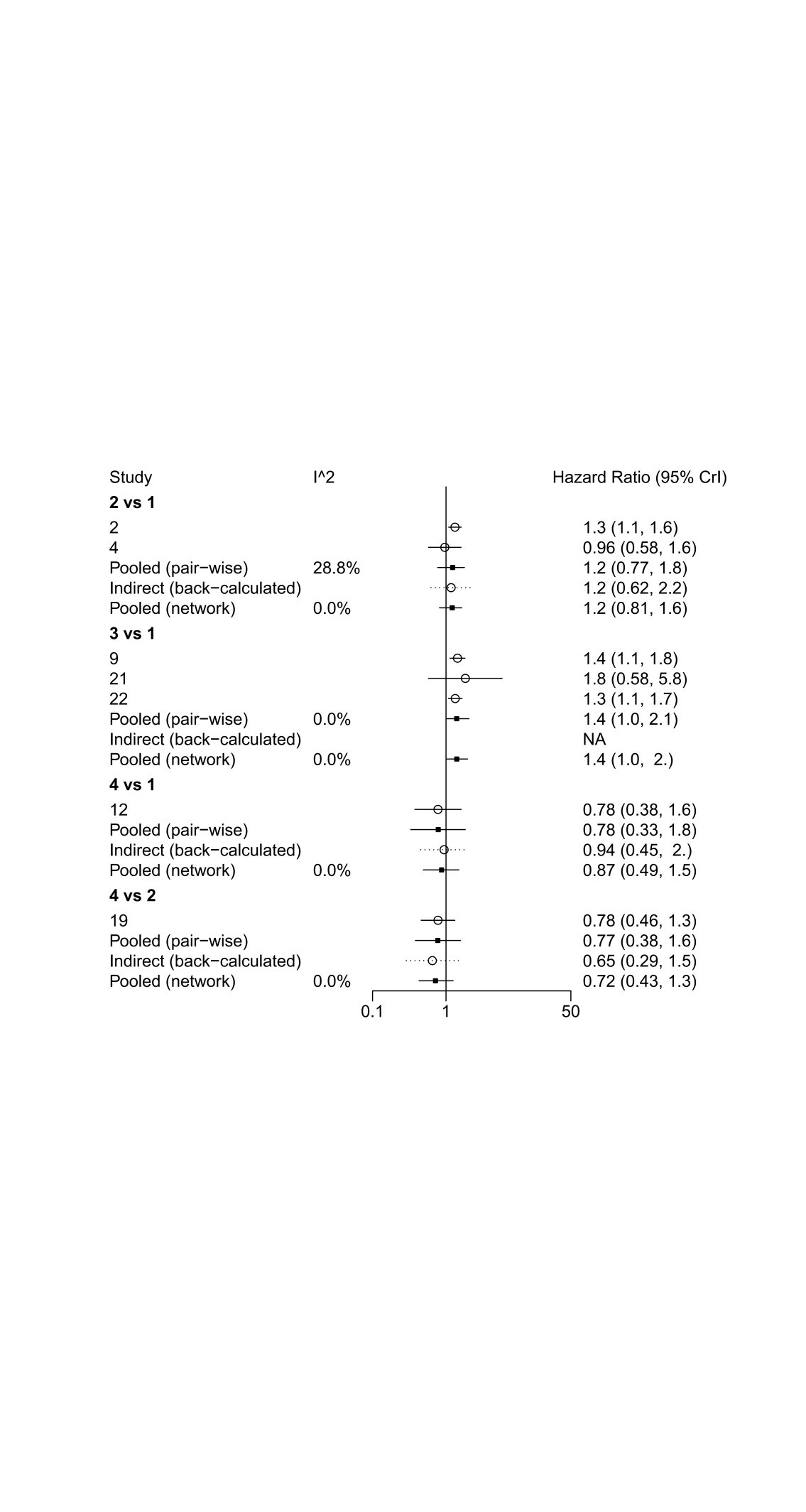


Heterogeneity of PFS After Excluding High Risk of Bias Studies

Note: In each figure, the bolded numbers represent specific treatment regimens, while the non-bolded numbers correspond to the original studies. 1: Taxanes + Platinum-based + 5-Fluorouracil; 2: Taxanes + Platinum-based; 3: Platinum-based + 5-Fluorouracil; 4: Targeted Therapy + Chemotherapy.


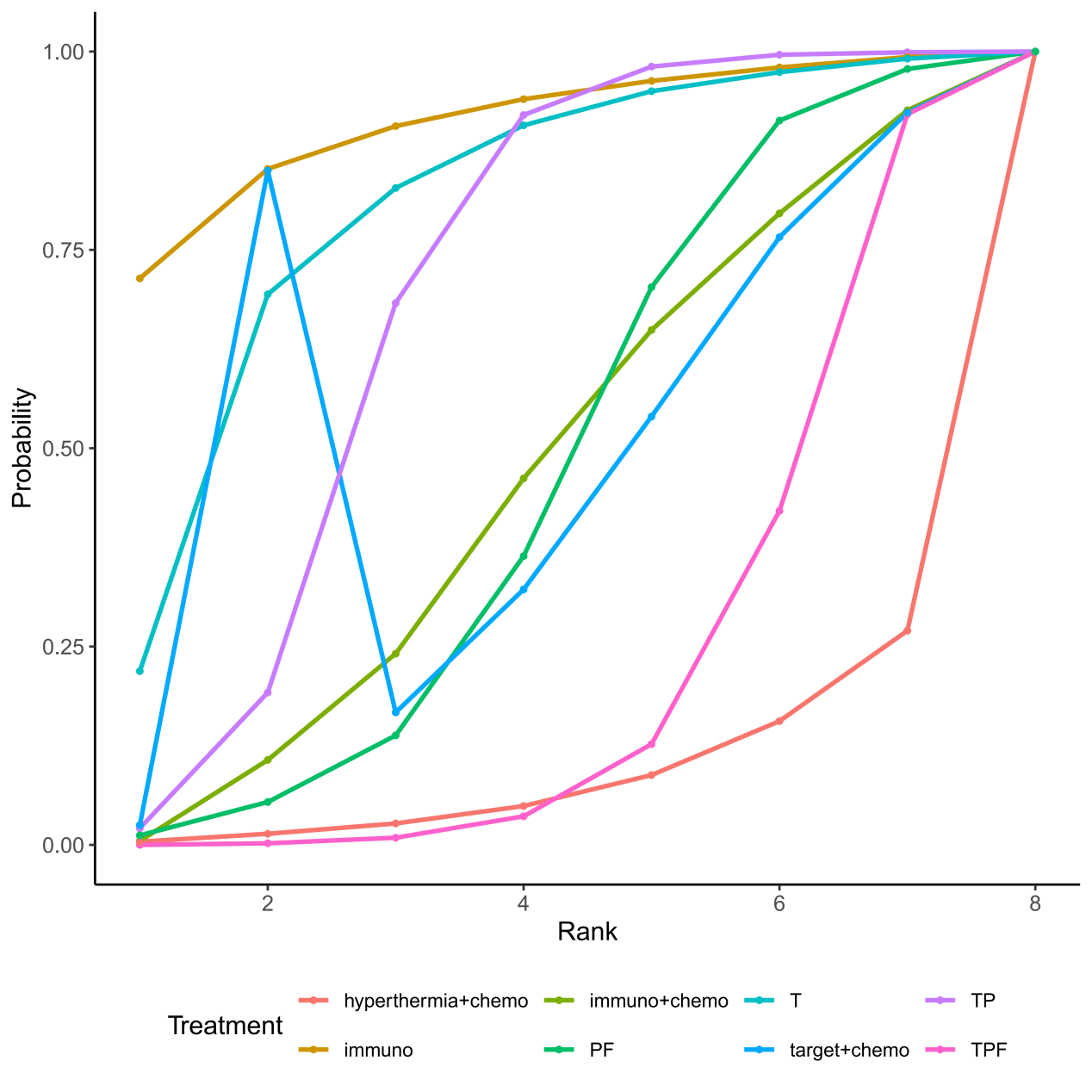


SUCRA for SAEs After Excluding High Risk of Bias Studies

Note: PF: Platinum-based + 5-Fluorouracil; TPF: Taxanes + Platinum-based + 5-Fluorouracil; TP: Taxanes + Platinum-based; immuno: Immunotherapy; immuno+Chemo: Immunotherapy + Chemotherapy; target+Chemo: Targeted Therapy + Chemotherapy; hyperthermia+Chemo: Hyperthermia + Chemotherapy; T: Taxanes.


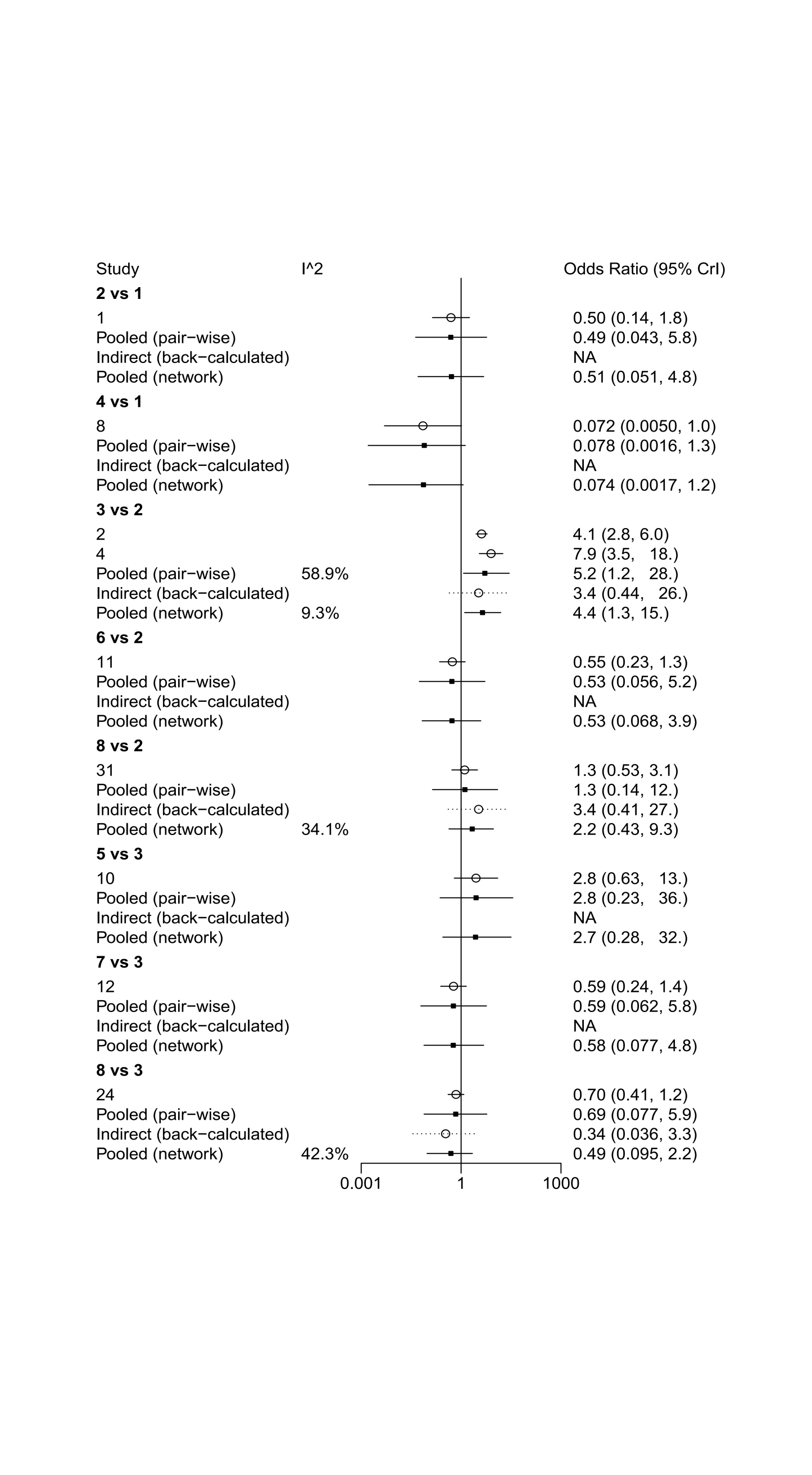


C

Heterogeneity of SAEs After Excluding High Risk of Bias Studies

Note: In each figure, the bolded numbers represent specific treatment regimens, while the non-bolded numbers correspond to the original studies. 1: Immunotherapy + Chemotherapy; 2: Taxanes + Platinum-based; 3: Taxanes + Platinum-based + 5-Fluorouracil; 4: Immunotherapy; 5: Hyperthermia + Chemotherapy; 6: Taxanes; 7: Targeted Therapy + Chemotherapy; 8: Platinum-based + 5-Fluorouracil.
