# Supplementary material for: Efficacy and Safety of Different Neoadjuvant Treatment Regimens in Locally Advanced Squamous Head and Neck Cancer
Source: Cancer Rep (Hoboken). 2026 Jan 26;9(1):e70447. doi: 10.1002/cnr2.70447 (PMC12835624; doi:10.1002/cnr2.70447)
Supplement: Supplementary file 2 — Data S2: Supplementary file 2. [file CNR2-9-e70447-s002.docx]

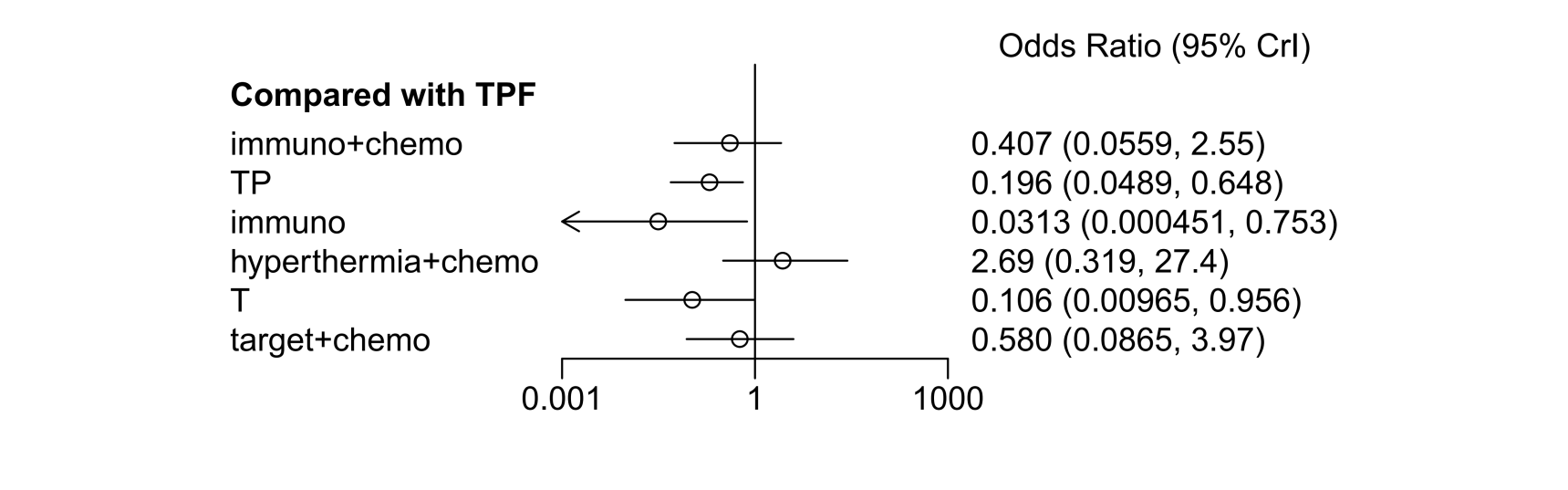


Analysis of Studies with SAEs Assessed by CTCAE 3.0 or Higher: A Forest Plot

Note: TPF: Taxane-based + Platinum-based + 5-Fluorouracil; TP: Taxane-based + Platinum-based; immuno: Immunotherapy; immuno+Chemo: Immunotherapy + Chemotherapy; target+Chemo: Targeted Therapy + Chemotherapy; hyperthermia+Chemo: Hyperthermia + Chemotherapy; T: Taxanes.

Differences with 95% confidence intervals not crossing 1 are statistically significant (*P* > 0.05).


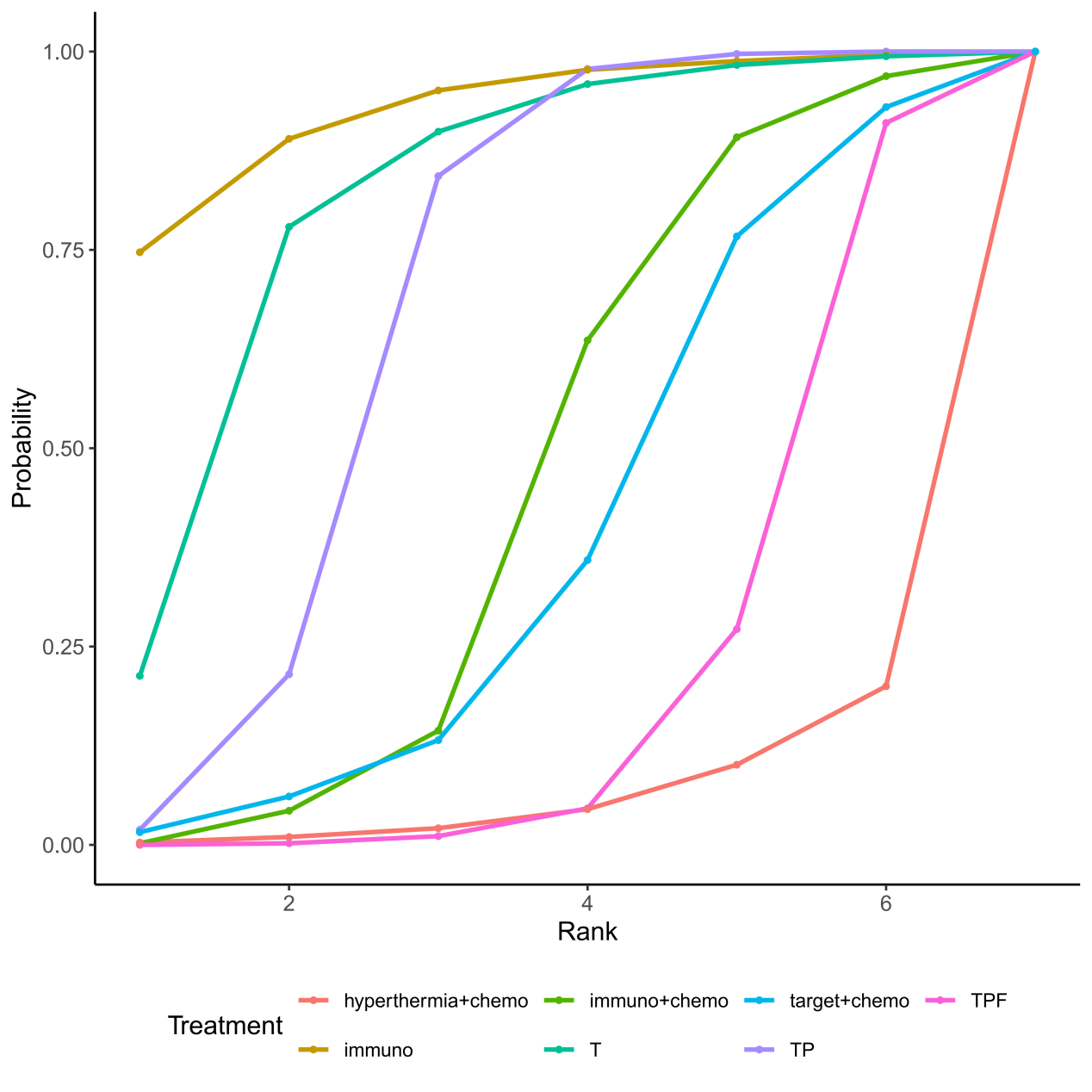


Analysis of Studies with SAEs Assessed by CTCAE 3.0 or Higher: A SUCRA Plot

Note: TPF: Taxane-based + Platinum-based + 5-Fluorouracil; TP: Taxane-based + Platinum-based; immuno: Immunotherapy; immuno+Chemo: Immunotherapy + Chemotherapy; target+Chemo: Targeted Therapy + Chemotherapy; hyperthermia+Chemo: Hyperthermia + Chemotherapy; T: Taxanes.
