# Supplementary material for: Efficacy and Safety of Different Neoadjuvant Treatment Regimens in Locally Advanced Squamous Head and Neck Cancer
Source: Cancer Rep (Hoboken). 2026 Jan 26;9(1):e70447. doi: 10.1002/cnr2.70447 (PMC12835624; doi:10.1002/cnr2.70447)
Supplement: Supplementary file 3 — Data S3: Supplementary file 3. [file CNR2-9-e70447-s001.docx]

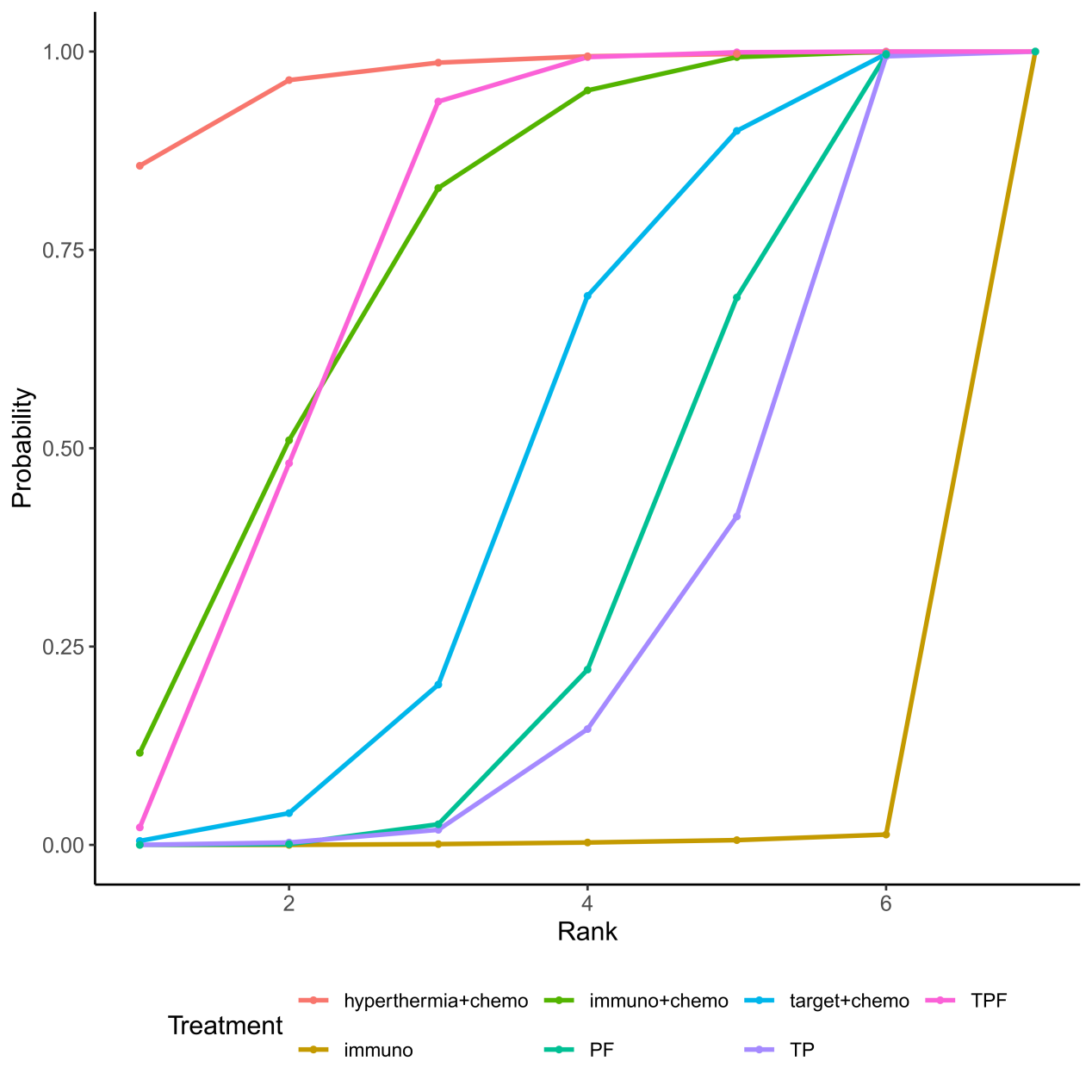


SUCRA for ORR after excluding NRS

Note: PF: Platinum-based + 5-Fluorouracil; TPF: Taxanes + Platinum-based + 5-Fluorouracil; TP: Taxanes + Platinum-based; immuno: Immunotherapy; immuno+Chemo: Immunotherapy + Chemotherapy; target+chemo: Targeted Therapy + Chemotherapy; hyperthermia+Chemo: Hyperthermia + Chemotherapy.


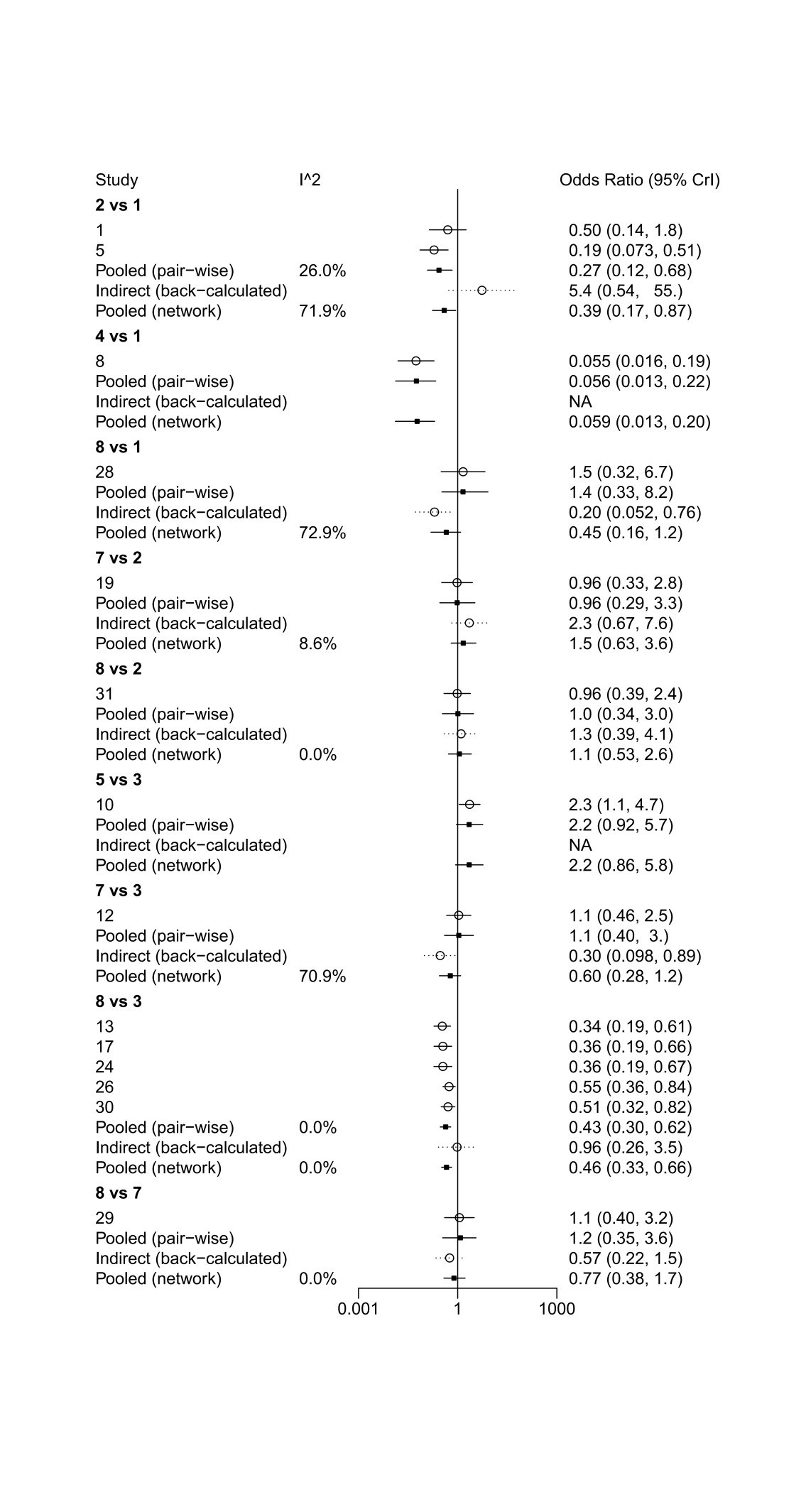


Heterogeneity in ORR after excluding NRS

Note: In each figure, the bolded numbers represent specific treatment regimens, while the non-bolded numbers correspond to the original studies. 1: Immunotherapy + Chemotherapy; 2: Taxanes + Platinum-based; 3: Taxanes + Platinum-based + 5-Fluorouracil; 4: Immunotherapy; 5: Hyperthermia + Chemotherapy; 7: Targeted Therapy + Chemotherapy; 8: Platinum-based + 5-Fluorouracil.


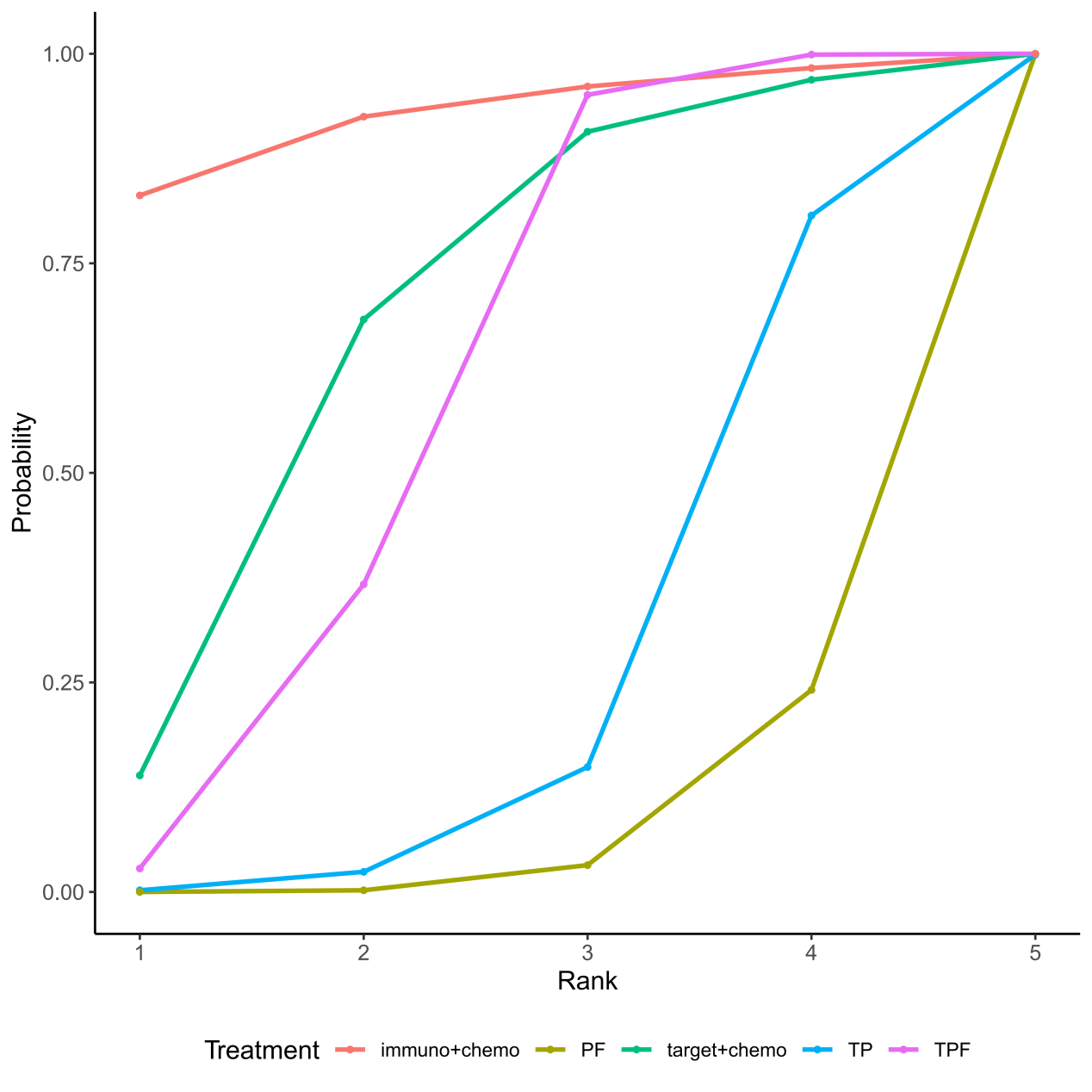


SUCRA for PFS after excluding NRS

Note: PF: Platinum-based + 5-Fluorouracil; TPF: Taxanes + Platinum-based + 5-Fluorouracil; TP: Taxanes + Platinum-based; immuno+chemo: Immunotherapy + Chemotherapy; target+Chemo: Targeted Therapy + Chemotherapy.


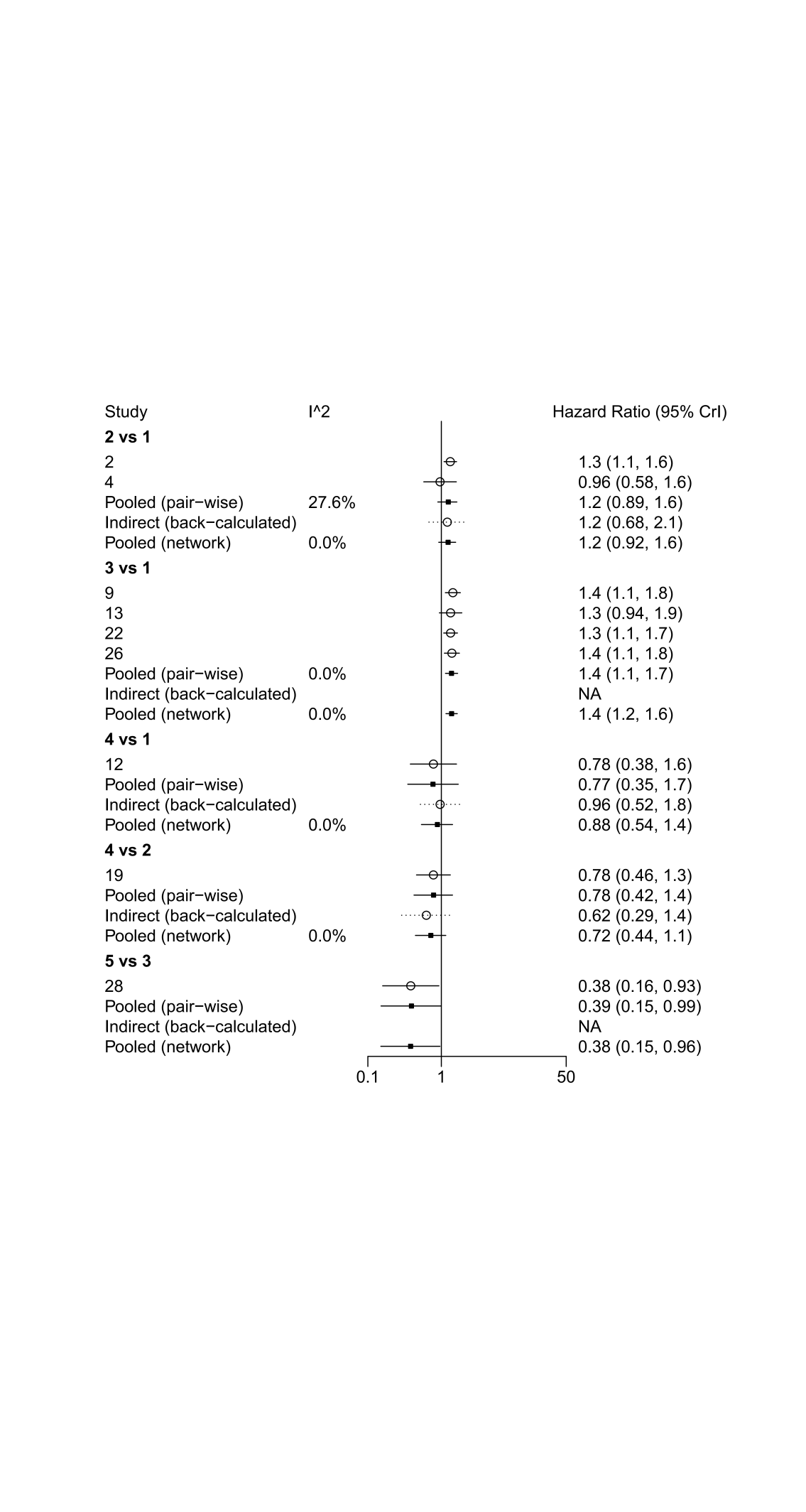


Heterogeneity in PFS after excluding NRS

Note: In each figure, the bolded numbers represent specific treatment regimens, while the non-bolded numbers correspond to the original studies. 1: Taxanes + Platinum-based + 5-Fluorouracil; 2: Taxanes + Platinum-based; 3: Platinum-based + 5-Fluorouracil; 4: Targeted Therapy + Chemotherapy; 5: Immunotherapy + Chemotherapy.


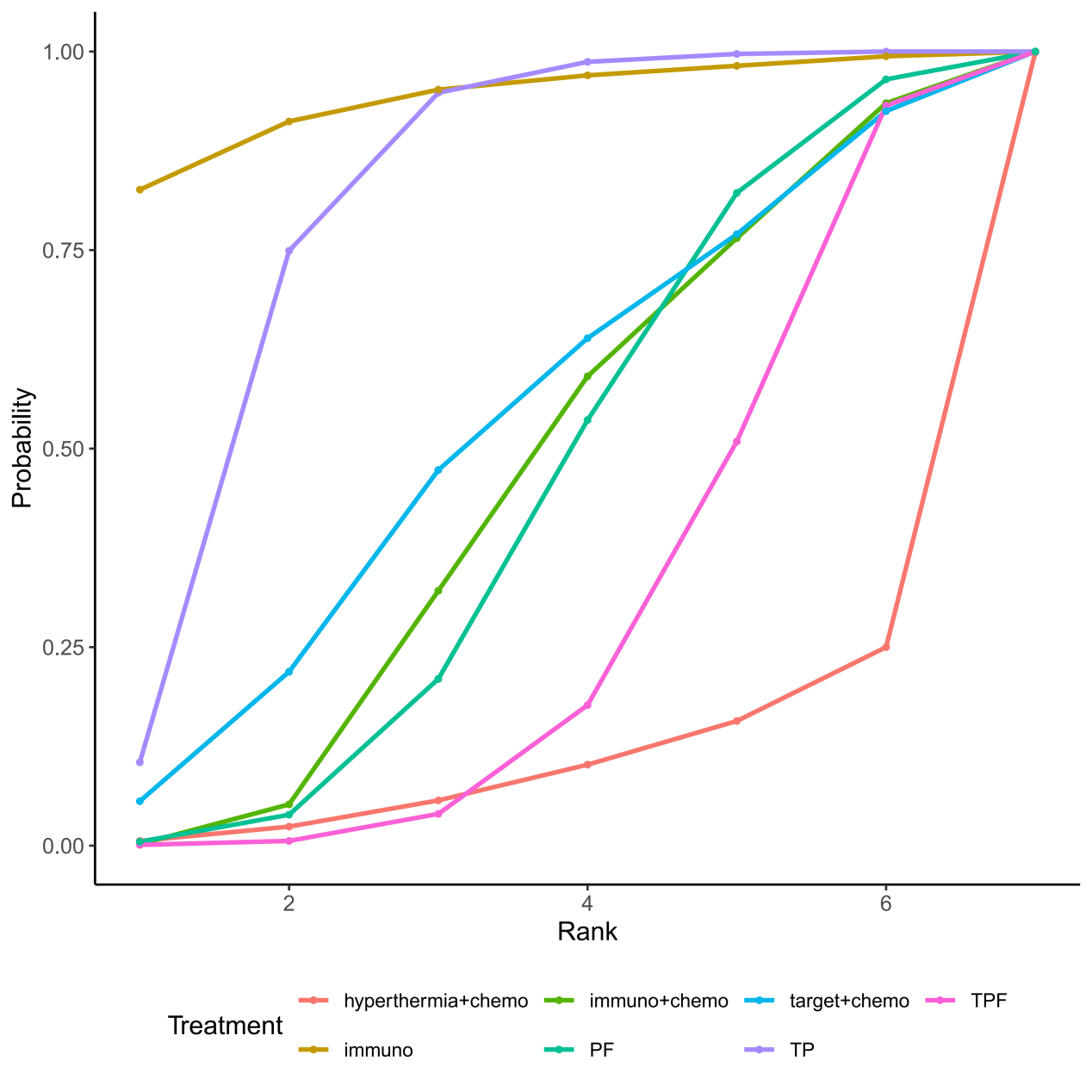


SUCRA for SAEs after excluding NRS

Note: PF: Platinum-based + 5-Fluorouracil; TPF: Taxanes + Platinum-based + 5-Fluorouracil; TP: Taxanes + Platinum-based; immuno: Immunotherapy; Immuno+Chemo: immunotherapy + Chemotherapy; target+Chemo: Targeted Therapy + Chemotherapy; hyperthermia+Chemo: Hyperthermia + Chemotherapy.


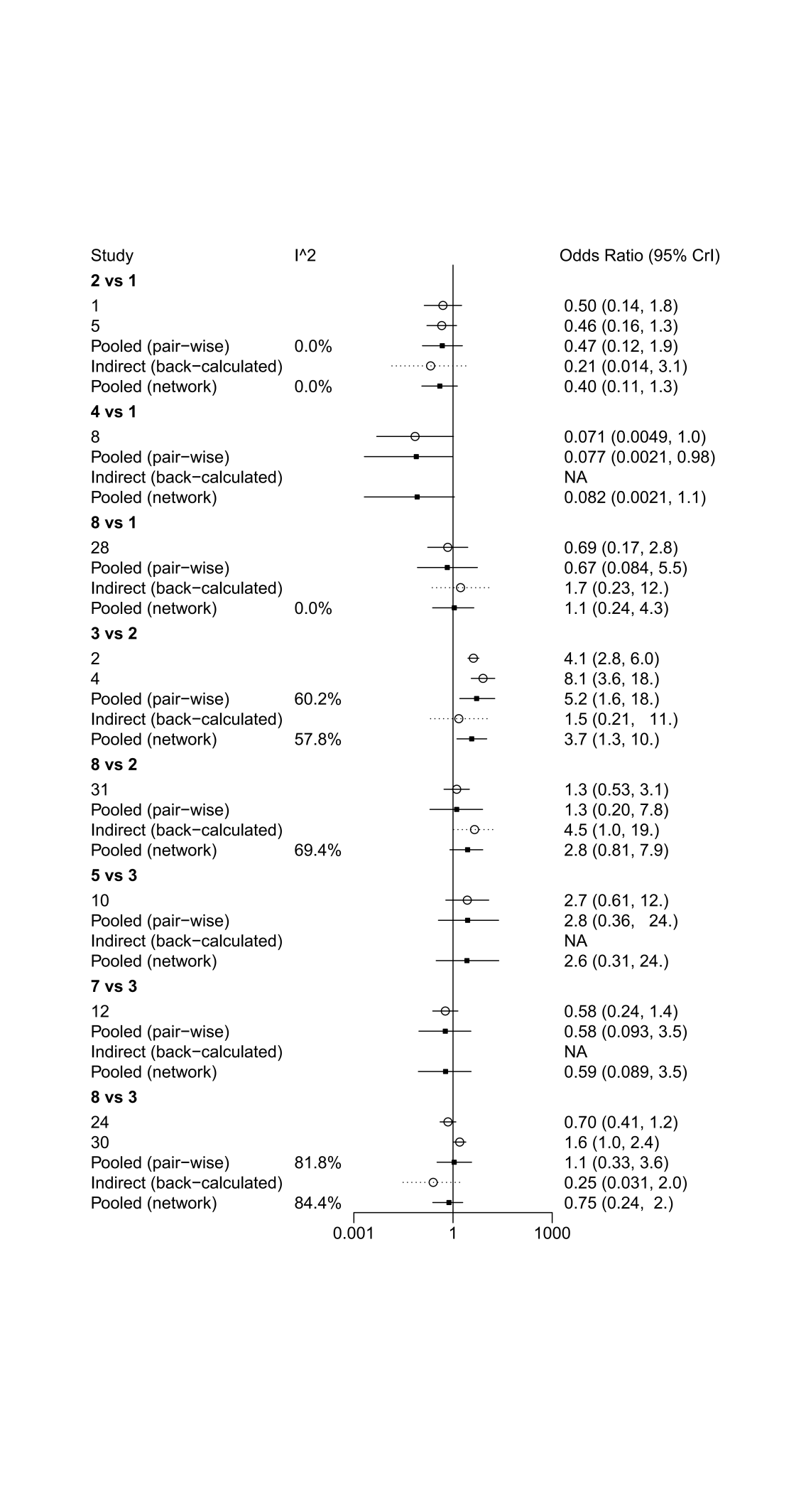


Heterogeneity in SAEs after excluding NRS

Note: In each figure, the bolded numbers represent specific treatment regimens, while the non-bolded numbers correspond to the original studies. 1: Immunotherapy + Chemotherapy; 2: Taxanes + Platinum-based; 3: Taxanes + Platinum-based + 5-Fluorouracil; 4: Immunotherapy; 5: Hyperthermia + Chemotherapy; 7: Targeted Therapy + Chemotherapy; 8: Platinum-based + 5-Fluorouracil.
